# Encoding of blink information via wireless contact lens for eye–machine interaction

**DOI:** 10.1093/nsr/nwaf338

**Published:** 2025-08-19

**Authors:** Haiqing Liu, Weijia Liu, Zhijian Du, Lifeng Wu, Minyan Chen, Zhiyi Gao, Kai Jiang, La Li, Zhiyong Fan, Guozhen Shen

**Affiliations:** School of Integrated Circuits and Electronics, Beijing Institute of Technology, Beijing 100081, China; School of Integrated Circuits and Electronics, Beijing Institute of Technology, Beijing 100081, China; School of Integrated Circuits and Electronics, Beijing Institute of Technology, Beijing 100081, China; School of Integrated Circuits and Electronics, Beijing Institute of Technology, Beijing 100081, China; School of Integrated Circuits and Electronics, Beijing Institute of Technology, Beijing 100081, China; CAS Key Laboratory of Magnetic Materials and Devices, Ningbo Institute of Materials Technology and Engineering, Chinese Academy of Sciences, Ningbo 315201, China; Chinese PLA General Hospital, Institute of Hepatobiliary Surgery of Chinese PLA, Key Laboratory of Digital Hepatobiliary Surgery, Beijing 100853, China; School of Integrated Circuits and Electronics, Beijing Institute of Technology, Beijing 100081, China; Department of Electronic & Computer Engineering, The Hong Kong University of Science and Technology, Hong Kong 999077, China; School of Integrated Circuits and Electronics, Beijing Institute of Technology, Beijing 100081, China

**Keywords:** eye–machine interaction, Ti_3_C_2_T*_x_* MXene, contact lens, wearable electronics, human–machine interface

## Abstract

Blinks controlled by ocular muscles and nerves can manifest as either involuntary physiological behaviors or volitional control actions, with the former serving spontaneous protective functions while the latter constitutes a biologically meaningful communicative signal. The encoding of blink information provides a novel eye–machine interaction (EMI) prototype within the realm of human–machine interaction, expanding human consciousness and capability boundaries. It facilitates motor and language rehabilitation, silent communication and even voluntary command execution. However, existing EMI devices face challenges related to wireless functionalities, ocular comfort and multi-route encoding/decoding orders. Here, we propose a wireless eye-wearable lens to encode conscious blink information via introduction of an RLC oscillating loop in the soft contact lens. The developed EMI contact lens incorporates a mechanosensitive capacitor, an inductive coil and the inherent loop resistance, generating characteristic resonance frequency for front-end capacitance signal transition or back-end control signal extraction. The EMI device delivers a sensitivity of 0.153 MHz/mmHg in the wide range of 0–70 mmHg for a normal intraocular pressure monitor and realizes conscious blink-based control command coding. A trial with participants having the EMI contact lens inserted demonstrates its wearability and biocompatibility. Finally, the five-route blink-based control command decoding mechanism is constructed via the EMI lens, linking blink counts to a drone's flight trajectory. The EMI contact lens offers an innovative prototype that transcends the capabilities of traditional brain–computer interfaces.

## Teaser

A wireless EMI contact lens enables real-time IOP monitoring and consciousness-blink-based drone control, demonstrating excellent biocompatibility and multi-route blink decoding for advanced human-machine interaction.

## INTRODUCTION

Sophisticated and precise eyes facilitate the connection between external stimuli and the complex subjective consciousness of the brain. This interaction opens new avenues for eye–machine interaction (EMI) that extend beyond traditional brain–computer interfaces (BCIs) for patients with quadriplegia [1,2], thereby redefining the boundaries of human consciousness and capability. Unlike a BCI based on electroencephalography, which needs complex visual- or auditory-evoked potential, algorithm, classification and electrical circuits to realize communication and control via brain activity, EMI accomplishes the command based on consciousness information generated from the brain via simple ocular movements, the accuracy of which reaches up to 100%, much higher than that of a BCI [3–5]. Ocular movements primarily include blinks and eye rotation [6]. Existing research on EMI devices that depend on eye rotation can be categorized into two groups: smart glasses with charge-coupled devices (CCDs) and a camera to record the eye rotation; and contact lenses with several metal coils. The former relies on redundant image collecting cards, control circuit, fixing devices and a computer for operation [7], while the latter requires at least four metal coils to track the eye rotation, resulting in reduced comfort and impaired vision [8].

Blinks offer several advantages as a form of ocular movement, including their visibility, stability, ease of capture, straightforward back-end processing algorithms and user-friendliness, especially when compared to eye rotation. Blinks can provide a comprehensive array of information, such as differences between the left and right eyes, blink count, duration of each blink and blinking frequency, enabling the generation of high-precision commands through blink encoding [9,10]. Statistical data reveal that a conscious blink will apply a pressure of approximately 30 mmHg on the cornea through the eyelid [11]. This suggests the blink-recording/transforming EMI devices can be fabricated by introducing a high-performance strain sensor based on a force-induced deformation mechanism in soft contact lenses, allowing for real-time intraocular pressure (IOP) monitoring, given the normal IOP ranges from 10 to 21 mmHg. The challenges in designing EMI contact lenses are mostly focused on: (i) achieving wireless EMI, while including near-field communication chips and metal coils that introduce rigidity to the soft lens; (ii) ensuring high sensitivity, for resistive-based contact lenses, which often struggle to eliminate wired signal transmission; and (iii) validating the encoding prototype of the EMI contact lens [12,13].

Here we report a wireless EMI contact lens by designing an RLC oscillating loop with a mechanosensitive capacitor, an inductive coil and the inherent loop resistance in the soft lens, realizing continuously monitored IOP and encoding the conscious blink information, like the duration of each blink, the blinking frequency, etc. Owing to the introduction of microstructures in an MXene-based capacitive sensor and inductive Al coil, the designed lens transfers the capacitance signal to characteristic resonance frequency and, as a result, delivers a sensitivity of 0.153 MHz/mmHg in the wide range of 0–70 mmHg for real-time IOP monitoring. The excellent biocompatibility of the EMI contact lens is demonstrated by *in*  *vivo* experiments on rabbit eyes and its wearability is verified in human eyes by several participants. Moreover, the EMI lens's encoding capability for conscious blink numbers is achieved by the blink-based control command decoding mechanism, and the demonstration of a five-route drone's flight trajectory further validates the possibility of the EMI, which can be applied in motor and language rehabilitation, and enhancement of human capabilities.

## RESULTS AND DISCUSSION

### Design of the EMI system

**Figure 1.**
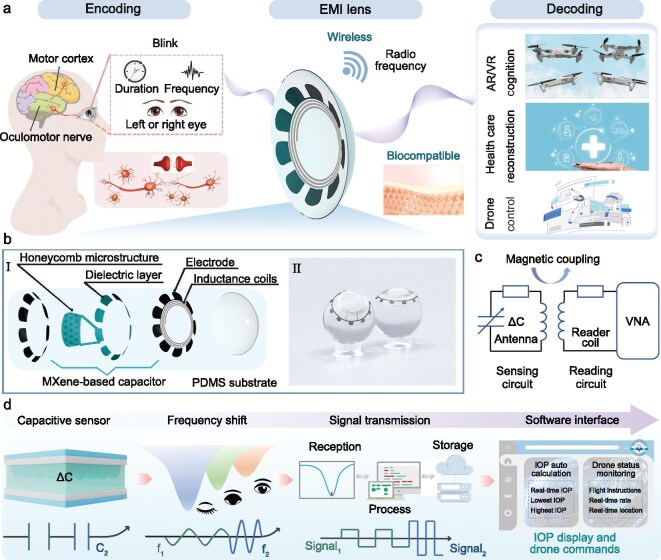
Design of the EMI system. (a) Left: encoding of blink information; the schematic indicates that the brain generates specific commands and stimulates the eyes to blink accordingly when a specific situation is encountered. Centre: EMI lens. Right: decoding the blink information for machine interface, healthcare and augmented reality (AR)/virtual reality (VR). (b) (I) The exploded structure diagram of the blink-recognizable EMI lens; (II) digital photographs of the EMI lens in different orientations. (c) The internal circuit of the EMI lens and the reading circuit of the frequency signal. (d) The encoding and decoding process of blink information. The capacitance of the sensor in designed EMI lens changes when the eyes switch between different states (eyes open, squinting and eyes closed) and then convert to frequency, which is recorded by the external coil and transmitted to the electrical circles for signal process, finally decoding for the drone control application.

It is widely accepted that the human eye represents a sophisticated biological interface capable of receiving and processing visual stimuli from the external environment and subsequently transmitting complex neurological information to the brain [14]. Furthermore, by leveraging advanced human–computer interaction technologies, the eye can also effectively communicate intricate subjective consciousness to the external environment [15–19]. To achieve precise and expeditious transmission of volitional commands​, we have proposed a highly sensitive soft smart contact lens by optimizing the device structure and control scheme, thereby establishing an EMI system for facilitating the control of external coded objects (Fig. 1). The visual system modulates blink dynamics according to eye-controlled coded objects, producing distinct ocular responses that correspond to specific operational requirements [20–22]. The EMI contact lens we designed not only deforms with the cornea and senses changes in the radius of curvature of the cornea due to changes in IOP (10–21 mmHg), but also monitors the blinking behavior of the wearer by detecting the pressure of the eyelid on the cornea (approximately 30 mmHg) [23]. Figure 1b shows the structure of each functional layer in the EMI lens, including the polydimethylsiloxane substrate, two MXene electrode layers, microstructured dielectric layer and an inductor coil, where the two-layer MXene electrodes and the microstructured dielectric layer together form a mechanosensitive capacitor. The EMI lens is advantageous due to its softness, thinness, lightness and transparency (Fig. 1b Ⅱ), fulfilling the requirements for controlling externally programmable objects without obstructing the field of view or causing damage to the cornea.

The RLC oscillating loop (Fig. 1c), consisting of a mechanosensitive capacitor, an inductive coil and the inherent loop resistance, can generate characteristic resonance frequency for back-end control signal extraction. A vector network analyzer (VNA) can obtain the reflection coefficient (S_11_) curves through a reading coil, which enables wireless measurement of the resonance frequency of the EMI lens under dynamic pressure conditions. The real part of the equivalent impedance of the VNA input port Z_1_ is as follows:


(1) \begin{eqnarray*}
{\mathrm{Re}}( {{Z_1}}) = {R_1} + 2\pi f{L_1}{k^2}Q\frac{{\frac{f}{{{f_2}}}}}{{1 + {Q^2}{{\left( {\frac{f}{{{f_2}}} - \frac{{{f_2}}}{f}} \right)}^2}}},
\end{eqnarray*}

Here, *R*_1_ and *L*_1_ represent the resistance and inductance of the reading coil, *f*_2_ and *Q* are the resonant frequency and quality factor of the EMI lens loop, *f* is the excitation frequency, and k stands for the coupling coefficient between the reading coil and the sensing coil. In addition, S_11_, Z_1_ and characteristic impedance of VNA Z_0_ have the following relationship:


(2) \begin{eqnarray*}
{S_{11}} = \frac{{{Z_1} - {Z_0}}}{{{Z_1} + {Z_0}}}.
\end{eqnarray*}

As the eye state changes from open to closed, the S_11_ parameters acquired by the VNA show an amplitude modulation and spectral perturbation. Through intelligent image recognition and algorithmic conversion, the resonance frequencies of the EMI lens are instantaneously captured and converted into corresponding pressure data, which are then seamlessly mapped to the cloud (Fig. 1d). Within the threshold range of IOP (5–25 mmHg), pressure data are presented in the dedicated IOP diagnostic module, and the system provides targeted diagnostic indicators. Beyond this range (>25 mmHg), pressure signals are converted by algorithm into drone navigation commands and are subsequently visualized and decoded within the drone control module of the software interface.

### The structural design and performance comparison of the EMI lens

**Figure 2.**
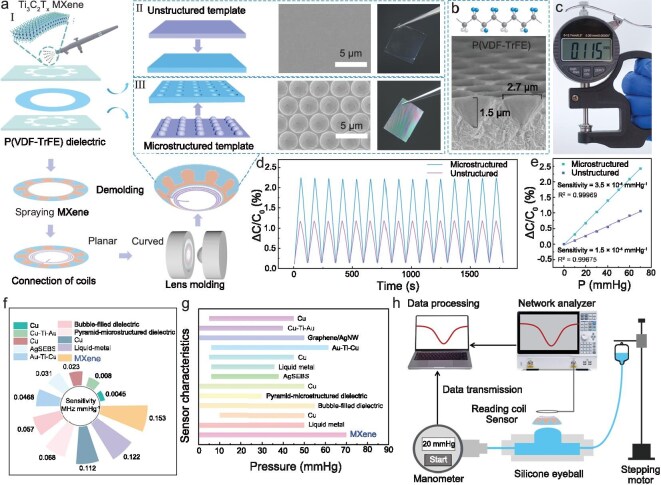
The structural design and performance comparison of the EMI lens. (a) Flow chart for the fabrication of the EMI lens (I). (II) and (III) show the preparation process, scanning electron microscopy (SEM) image and physical photograph of the unstructured and microstructured dielectrics, respectively. (b) Parametric characterization of the EMI lens: the molecular structure diagram of P(VDF-TrFE) and the 3D SEM image of the microstructured dielectric. (c) Characterization of the overall thickness of the EMI lens. (d) Ratio of change in capacitance to initial value (ΔC/C_0_) of microstructured and unstructured dielectric-sensitive capacitors during cycling in the pressure range of 0–70 mmHg. (e) Linear fitting curves of ΔC/C_0_ versus IOP for mechanosensitive capacitors with microstructured and unstructured dielectric. Comparison of measurement range (f) and sensitivity (g) of previously reported corneal contact lens-based IOP sensors with the EMI lens. (h) Schematic diagram of the analog IOP testing platform, which contains three modules: the analog eyeball, the pressure adjustment system, and the frequency signal testing system.

Figure 2a illustrates the device fabrication process, where the microstructured dielectric is first prepared by inverse molding. Ti_3_C_2_T*_x_* MXene electrodes are sprayed on both sides of the dielectric using a mask plate, followed by electrically connecting the inductive coil fabricated by a laser marking technique to form a complete RLC loop. Finally the EMI lens is assembled after encapsulation and curvature modification. The mechanosensitive capacitor is designed in a petal shape (Fig. S1), which helps to uniformly distribute the mechanical stress, reducing damage to the electrode during the curvature stage. In addition, the capacitor is positioned at the corneal–scleral junction (approximately 9–13 μm), an area of thin and soft tissue that is sensitive to tiny pressure changes [24]. Ti_3_C_2_T*_x_* MXene has superior conductivity, excellent mechanical flexibility and favorable biocompatibility, with a transverse size of more than 3 μm, which renders it an ideal material for use as a capacitive electrode (detailed synthesis of the Ti_3_C_2_T*_x_* MXene is described in Note S1 and Figs S2–S7) [25,26]. In terms of the dielectric, poly(vinylidene fluoride-*co*-trifluoroethylene) [P(VDF-TrFE)] layers not only have excellent flexibility but also feature high dielectric constants and low dielectric losses [27,28]. By changing the pattern, spacing and arrangement of the petals, we also prepared various capacitors (Fig. S12) to verify the feasibility of mass production. To improve the pressure detection sensitivity of the EMI lens, a honeycomb microstructure was fabricated in the dielectric layer using the inverted template method to introduce the air medium [29,30]. Compared to the transparency of planar dielectric films (Fig. 2a Ⅱ), microstructured dielectric films exhibit significant multi-color optical effects resulting from the selective reflection and diffraction behavior of incident light at the uniformly sized and directionally ordered micropore arrays on the surface (Fig. 2a Ⅱ and Fig. S8) [31]. The cross-sectional morphology further quantitatively characterizes the geometry of the micropores, revealing a pore diameter of 2.7 μm and a depth of 1.5 μm (Fig. 2b). Figure 2c shows that the thickness of the EMI lens is 115 μm, which is comparable to that of commercial contact lenses, ensuring that there is no noticeable foreign body sensation when worn.

To verify the effect of the honeycomb microstructure, pressure responses of the capacitors with two dielectric structures were investigated using a high-precision LCR meter. As shown in Fig. 2d, after 15 consecutive pressure cycling experiments in the pressure range of 0–70 mmHg, we observed that the capacitance of the capacitor with a honeycomb microstructure has a significantly larger change in amplitude, which is more than double that of the capacitor without microstructure. From the regression analysis in Fig. 2e, it can be seen that the response of both mechanosensitive capacitors to pressure indicates good linearity (linear regression coefficients, R^2^ = 0.9997 and 0.9967), whereas the capacitor with honeycomb microstructure presents a higher sensitivity of 0.232 pF/mmHg during the pressure increase. Based on the above structural design and material optimization, the prepared EMI lens has obvious advantages over existing IOP sensors reported in the literature in terms of key metrics (Fig. 2f and g and Tables S1–S2): the sensitivity is as high as 0.153 MHz/mmHg, and the range of pressure detection covers 0–70 mmHg. Both capacitance response and frequency response tests are performed on a simulated IOP test platform, as shown in Fig. 2h, which mainly consists of a bionic eyeball, a pressure regulation system and a signal testing system (high-precision LCR meter or VNA) (Note S6 and Fig. S17).

### Performance characterization and mechanism exploration of mechanosensitive capacitor

**Figure 3.**
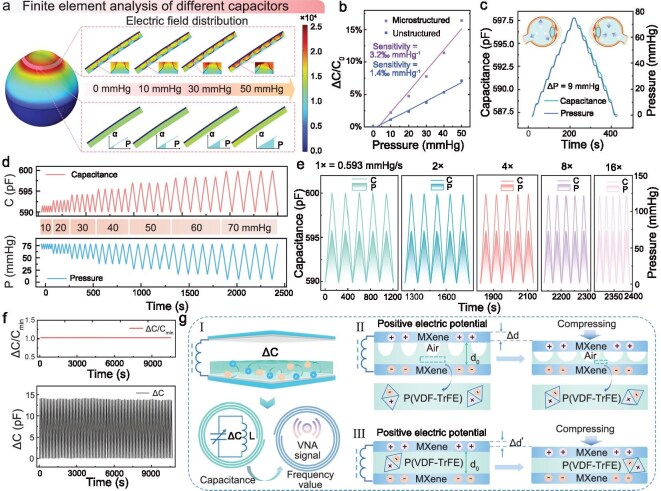
Performance characterization and mechanism exploration of mechanosensitive capacitor. (a) Electric field distribution of capacitors with microstructured and unstructured dielectrics on the eyeball at different pressures (0, 30, 50, 70 mmHg) in finite element simulations. (b) Linear fitting curves of ΔC/C_0_ versus IOP for capacitors with microstructured and unstructured dielectrics in finite element simulations. (c) Capacitance response as IOP rises and falls in steps of 9 mmHg. (d) Capacitance response curves when IOP is cycled in different ranges (0–10, 0–20, 0–30, 0–40, 0–50, 0–60, 0–70 mmHg). (e) Capacitance response curves when the IOP was cycled at different speeds (1×, 2×, 4×, 8×, 16×) in the range of 0–70 mmHg. (f) ΔC/C_0_ curve (top) within each cycle and ΔC curve (bottom) for mechanosensitive capacitor, with pressures cycling in the 0–70 mmHg range for more than 3 h. (g) Mechanistic investigation of the variation of capacitances with pressure for capacitors with microstructured and unstructured dielectrics.

To comprehensively investigate and evaluate the feasibility and performance of the designed EMI lens structure, finite element analysis (FEA) simulations were conducted on the strain characteristics of the eye and the dynamic response mechanisms of the different structural mechanosensitive capacitors to the pressure variations (Fig. 3a, Note S3 and Figs S9–S11). The results reveal that the corneal–scleral junction exhibits the most significant geometrical deformation features under application of the elevated IOPs, and it is noteworthy that this region is the primary localization region of the mechanosensitive capacitor. Giving a precise potential (1 V) between the poles of the mechanosensitive capacitors, quantitative analysis of the electric field distribution was conducted. Encouragingly, complex field-intensity profiles with non-linear evolution were observed upon introduction of microstructured dielectrics, suggesting improved sensitivity of capacitance variations. The capacitance–pressure curve, depicted in Fig. 3b, explicitly reveals that the sensitivity of the mechanosensitive capacitor is enhanced by over 2-fold compared to the original capacitor after microstructure optimization.

Considering that IOP exhibits susceptive dynamic fluctuations [32], and that human eyes frequently engage in irregular blinking and eye movements, the EMI lens is expected to provide adequate measurement sensitivity and precise dynamic response to capture such complex physiological parameter changes in real time. Figure 3c indicates that the mechanosensitive capacitor presents a precise stepwise response as the pressure steps up and down over the pressure range of 0–70 mmHg. As shown in Fig. 3d, capacitance of the mechanosensitive capacitor demonstrates remarkable linear tracking capability in cyclic tests over diverse pressure ranges (from 0–10 to 0–70 mmHg), capturing subtle changes in pressure without delay. Further validation indicates that, based on a 1× benchmark rate (0.593 mmHg/s), the capacitances maintain an exceedingly consistent cycling characteristic when varying IOP within the range of 0–70 mmHg at different rates of 2×, 4×, 8×, 16× etc. This is illustrated in Fig. 3e, which highlights the dynamic adaptability and accuracy of the mechanosensitive capacitor. In practice, we also have implemented 3-h fatigue tests (Fig. 3f), which indicate that there is outstanding stability of the device to cope with the continuing utilization. Two time periods are randomly selected to show individual waveforms in a long-time loop (Fig. S13), which are favorably smooth and repeatable. Additionally, Figs S14–S15 present the consistent ΔC/C_0_ value by simultaneously evaluating multiple mechanosensitive capacitors, confirming the homogeneity in the fabrication process and electrical characteristics of the devices.

Figure 3g further elaborates on the microscopic mechanism and macroscopic response of pressure on the dielectric property of capacitor. Under the action of small external pressure, its capacitance exhibits an approximately linear relationship according to the formula ${\mathrm{C\; = \;\varepsilon S}}/{\mathrm{d}}$. For capacitors with a honeycomb microstructured dielectric, the pressure sensitivity is significantly higher than that of capacitors with ordinary structures, which stems from the following mechanism: (i) a significant change in the effective permittivity (ε) can be achieved due to the compressed air inside the honeycomb microstructured dielectric; and (ii) the introduction of honeycomb microstructures results in a prominent diminution of Young's modulus of the dielectric layer, and thus the change in the thickness (Δd) of the dielectric layer under the action of pressure is more substantial than Δd′ [33].

### Frequency response and biocompatibility validation of the EMI lens

**Figure 4.**
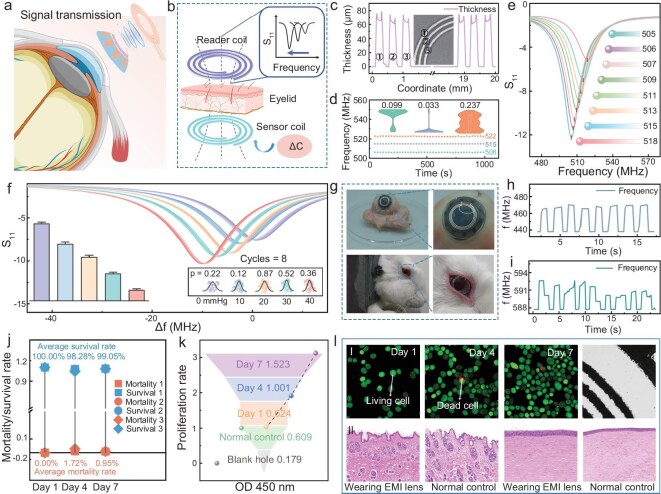
Frequency response and biocompatibility validation of the EMI lens. (a) Schematic of signal transmission of the EMI lens worn on the cornea. (b) Flow of capacitive to frequency signal conversion during signal transmission with eyes closed. (c) Sensor coil microscopy image, as well as step profilometer image. (d) Baseline frequency variation and error analysis of the three different EMI lenses over a cycle of 1000 s. (e) S_11_ curves read by the VNA as the pressure is increased from 0 to 70 mmHg (in 10 mmHg steps). The resonant frequencies of the EMI lenses at each pressure (0, 10, 20, 30, 40, 50, 60, 70 mmHg) are labeled from top to bottom. (f) S_11_ curves read by the VNA for each pressure (0, 10, 20, 30, 40 mmHg) when the pressure is cycled eight times in the range of 10–50 mmHg. (g) Digital photographs during testing in the isolated porcine eye and live rabbit eye. (h) The frequency response of the EMI lens during blinking in the isolated porcine eye. (i) Frequency response of the EMI lens during blinking in the rabbit eye. (j) Mortality and survival statistics in cytotoxicity testing. (k) OD value and proliferation rate statistics in cytotoxicity testing. (l) (I) illustrates microscopic images of cells and the EMI lens at different days of co-culture (1, 4, 7 days), while (II) shows the eyelid and corneal tissues of rabbits after 3 days of wearing the EMI lens.

In the circuit structure of the EMI lens, the pressure information is encoded in the slight alteration of capacitance, and the efficient transmission of pressure information can be realized through the electromagnetic coupling mechanism between the internal sensing coil and the external reading coil (Fig. 4a). When the eyelid closes, the mechanosensitive capacitor is compressed, leading to an increase of its capacitance. This change further causes a decrease in the resonance frequency of the LCR loop, which is ultimately reflected in the characteristic frequency shift of the S_11_ parameter curve of the VNA (Fig. 4b). Figure 4c shows the structural features of the three-turn aluminum coil with a flat surface (65 μm thickness), uniform spacing (200 μm) and consistent linewidth (200 μm). The continuous performance test on the three EMI lenses for a duration of 1000 s, as shown in Fig. 4d, demonstrates that the baseline frequencies exhibit significant stability (standard deviation of 0.033, 0.099 and 0.237, respectively) due to the absence of frequency drift or fluctuation. Figure 4e reflects a correlation between pressure and resonance frequency; as pressure gradually increases from 0 to 70 mmHg, the resonance frequency of the device exhibits an obvious linear decreasing trend, with a linear correlation coefficient (R^2^) of 0.9793 and a sensitivity of 0.186 MHz/mmHg (Fig. S25). Nevertheless, the pressure range of 0–40 mmHg was already sufficient for the application of the EMI lens, therefore eight consecutive pressure cycling tests were performed in this range. Promisingly, as shown in Fig. 4f, the resonance frequency of the EMI lens exhibits dramatic repeatability at each stable pressure point (0, 10, 20, 30 and 40 mmHg), with distinct differences in resonance frequencies at various pressure points that are easily discernible. Figure S26 presents an infrared thermogram of the EMI lens under operating conditions, clearly depicting the surface temperature distribution of the lens and confirming its thermal safety in the physiological temperature range.

Three different biological models were selected to validate the functionality of the EMI lens as an EMI: an isolated porcine eye, an *in vivo* rabbit eye and an artificial eyeball model. These models were used to evaluate the response characteristics of the EMI lens to blinking behavior. As shown in Fig. 4g and Fig. S33a, the EMI lenses exhibit a favorable fit on the isolated porcine eye, rabbit eye and eyeball model, without disengaging with eye movements. In parallel, the frequency response (Fig. 4h and i and Fig. S33b) indicates that, although subject to complex physiological conditions, there is a detectable and striking decrease in the resonance frequency of the EMI lens during the eyelid closure phase across all models. Notably, the EMI lens achieves a system-level temporal resolution of 181 ms (distinct from sensor-level resolution, Fig. S35), which is sufficient for detecting conscious blinks given their characteristic duration exceeding 0.7 s.

To ensure whether the EMI lens is secure for regular wear, its biocompatibility was rigorously evaluated by a 7-day cytotoxicity assay using human umbilical vein endothelial cells (HUVEC) as a standard model, which can be indefinitely passaged. As illustrated in Fig. 4l I and Figs S29–S30, the HUVEC cells were uniformly distributed on the surface of the device during the 7 days of co-cultivation with the EMI lenses, with only sporadic dead cells observed. Furthermore, the cell survival rate remained above 98% throughout the incubation process (Fig. 4j, Table S3), while the cell proliferation rate reached 312.6% on Day 7 (Fig. 4k, Table S4), indicating negligible toxicity of the EMI lenses. In addition, the EMI lens was also worn on the eyes of rabbits for 3 consecutive days. Daily ocular slit-lamp and white light photographs showed that the eyes of rabbits did not suffer any obvious damage (Figs S27–S28). After 3 days, corneal and eyelid tissue sections were taken post-euthanasia of the rabbits for hematoxylin and eosin (H&E) staining observation (Fig. 4l II), revealing no significant alterations in the cellular morphology and structure of the rabbit eye tissues before and after lens wearing. In summary, the EMI lens can sensitively detect IOP and blinking behavior and has exceptional biocompatibility to accommodate an EMI system.

### EMI applications

**Figure 5.**
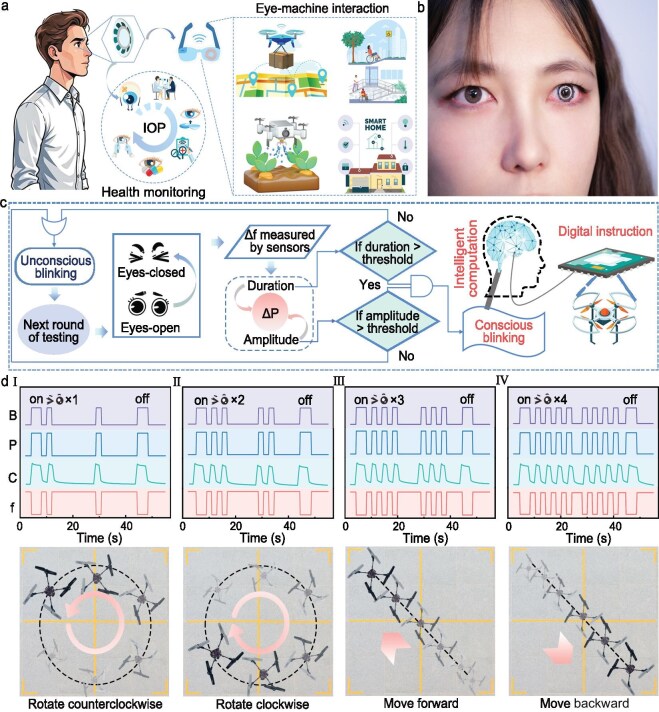
EMI applications. (a) Different application scenarios of the EMI lens include both health monitoring and EMI. (b) Digital photograph of the EMI lens actually worn in the human eye. (c) Recognition of conscious and unconscious blinks and generation of drone control commands. (d) (I–IV) represent the signal waveform graphs and drone flight trajectories when the drone rotates clockwise, counterclockwise, forward and backward, respectively. In the signal waveform graphs, the purple, blue, green and red curves indicate whether the model or rabbit blinks, the measured value of pressure, the capacitance of the mechanosensitive capacitor and the resonance frequency of the EMI lens, respectively.

Long-term, wireless real-time monitoring of IOP and eye motions can be realized by precisely integrating the reading coil into the eyeglass lens and embedding the signal transmission circuitry into the frame, aided by modern mobile software algorithms (Fig. 5a) [34]. For patients with eye diseases, continuous IOP monitoring is of great clinical significance. By obtaining IOP data in real time, medical professionals can accurately assess the state of eye health and decide promptly on the need for medication or surgery [32]. Remarkably, the EMI system based on the EMI lens has shown impressive application prospects in various fields. Potential application scenarios include, but are not limited to, precise material delivery by drones in disaster areas, intelligent irrigation systems by agricultural drones, eye-controlled device interaction in smart home environments (e.g. lighting systems and door lock control) and assisted mobility technology for people with severe physical disabilities or amyotrophic lateral sclerosis (ALS). After strict disinfection and soaking in commercial contact lens care solution for 10 min, the EMI lens was successfully worn by the subject (Fig. 5b), with no obvious physiological rejection or discomfort symptoms observed through half an hour of continuous wearing, validating the use of the EMI lens for practical applications.

The human eye achieves a physiological regulatory mechanism of corneal wetting, cleaning and fatigue relief through an unconscious blinking action of 10–20 times/min [35]. This physiological process, controlled by the autonomic nervous system, also induces tiny deformations in the EMI lens, leading to minor fluctuations in the resonance frequency. Compared to unconscious blinking, conscious blinking exhibits striking physiological differences such as significantly increased eyelid pressure on the cornea and prolonged duration of closure (conscious blinking is typically >0.7 s, and unconscious blinking is <0.4 s). Based on this physiological feature, the signal processing algorithm detects the amplitude (A) and duration (T) of the frequency fluctuation to realize the precise recognition of blink type (Fig. 5c); when A or T is below the preset threshold, it is judged as an unconscious blink and ignored; when both A and T exceed the threshold, it is recognized as a conscious blink, and is converted into an executable and precise control command. In the experiment for distinguishing between conscious and unconscious blinking, the system demonstrated accurate detection capability throughout the 1-h test period, with no observed false-positive results (Fig. S34).

To test the feasibility of the EMI system in real-world application scenarios, a drone is selected as a programmable object for concrete control experiments as its complex multi-dimensional motion state provides a representative experimental platform for system validation. A blink-based control command decoding mechanism is developed, mapping blinks to flight commands as follows: one blink triggers counterclockwise rotation, two blinks trigger clockwise rotation, three blinks control the forward movement of the drone, four blinks control the backward movement of the drone, and a long period of eye closure indicates the opening and closing of the control system. During the experiment, after the drone took off, the control system was activated, followed by the execution of successive blinks and observation of the drone's flight trajectory. It is encouraging that the capacitance and resonance frequency of the EMI lens exhibit significant changes when the eyelid is closed, while the drone flies purposefully in the limited space according to the preset commands (Fig. 5d and Movie S1), which verifies the practicality of the EMI system. To further validate the feasibility of the EMI system, a live rabbit was chosen as a representative sample of the authentic physiological system. In the experimental design, a quantitative correspondence between blinking time and flight maneuvers was constructed by accurately measuring the duration of the rabbit's blinking time: 1–2 s of blinking period corresponds to counterclockwise rotation of the drone, 2–3 s corresponds to clockwise rotation, 3–4 s corresponds to forward movement, and 4–5 s corresponds to backward movement. The results in Fig. S36 and Movie S2 demonstrate that the drone is on track to execute the preset flight trajectory accurately during the rabbit's sustained eye closure. After the experiment, the rabbits were assessed and found to maintain a normal level of locomotion and feeding behavior, with no obvious ocular abnormalities, indicating that the procedure did not affect the basic physiological functions of the subject animal.

## CONCLUSION

In summary, an EMI contact lens incorporating a mechanosensitive capacitor, an inductive coil and the inherent loop resistance has been developed to realize continuous IOP monitoring and conscious blink-based command coding. Based on the practical application scenarios of eye-controlled coded objects, the visual system can generate blink behaviors that meet specific command requirements, while the EMI lens enables precise recognition of these ocular responses. The designed wireless EMI contact lens with an RLC oscillating loop in the soft substrate generates characteristic resonance frequency for capacitance change under high IOP, achieving a sensitivity of 0.153 MHz/mmHg in the wide range of 0–70 mmHg for real-time IOP monitoring. The high cell survival rate of 98%, the cell proliferation rate of 312.6% on Day 7, the H&E staining test and *in*  *vivo* experiments on rabbit eyes demonstrate the excellent wearability and biocompatibility of the EMI contact lens, allowing the verification on human eyes. Furthermore, the EMI contact lens is applied in the drone's flight trajectory control via conscious blinking, establishing a five-route blink-based command decoding mechanism that maps blink counts to flight paths, offering a compelling concept for human–machine interaction.

## MATERIALS AND METHODS

Detailed materials and methods are available in the Supplementary data.

## Supplementary Material

nwaf338_Supplemental_Files

## References

[1] Wang Z, Shi N, Zhang Y et al. Conformal in-ear bioelectronics for visual and auditory brain-computer interfaces. Nat Commun 2023; 14: 4213. 10.1038/s41467-023-39814-6 37452047 PMC10349124

[2] Assi DS, Huang H, Karthikeyan V et al. Topological quantum switching enabled neuroelectronic synaptic modulators for brain computer interface. Adv Mater 2024; 36: 2306254. 10.1002/adma.202306254 38532608

[3] Karas K, Pozzi L, Pedrocchi A et al. Brain-computer interface for robot control with eye artifacts for assistive applications. Sci Rep 2023; 13: 17512. 10.1038/s41598-023-44645-y 37845318 PMC10579221

[4] Braun J-M, Fauth M, Berger M et al. A brain machine interface framework for exploring proactive control of smart environments. Sci Rep 2024; 14: 11054. 10.1038/s41598-024-60280-7 38744976 PMC11584623

[5] Zhou Y, Sun Z, Ding Y et al. An ultrawide field-of-view pinhole compound eye using hemispherical nanowire array for robot vision. Sci Robot 2024; 9: eadi8666. 10.1126/scirobotics.adi8666 38748782

[6] Wu EG, Brackbill N, Rhoades C et al. Fixational eye movements enhance the precision of visual information transmitted by the primate retina. Nat Commun 2024; 15: 7964. 10.1038/s41467-024-52304-7 39261491 PMC11390888

[7] Xiao W, Sharma S, Kreiman G et al. Feature-selective responses in macaque visual cortex follow eye movements during natural vision. Nat Neurosci 2024; 27: 1157–66. 10.1038/s41593-024-01631-5 38684892 PMC11156562

[8] Zhu H, Yang H, Xu S et al. Frequency-encoded eye tracking smart contact lens for human–machine interaction. Nat Commun 2024; 15: 3588. 10.1038/s41467-024-47851-y 38678013 PMC11055864

[9] Bae B, Lee D, Park M et al. Stereoscopic artificial compound eyes for spatiotemporal perception in three-dimensional space. Sci Robot 2024; 9: eadl3606. 10.1126/scirobotics.adl3606 38748779

[10] Yang B, Intoy J, Rucci M. Eye blinks as a visual processing stage. Proc Natl Acad Sci USA 2024; 121: e2310291121. 10.1073/pnas.2310291121 38564641 PMC11009678

[11] Ren X, Zhou Y, Lu F et al. Contact lens sensor with anti-jamming capability and high sensitivity for intraocular pressure monitoring. ACS Sens 2023; 8: 2691–701. 10.1021/acssensors.3c00542 37262351

[12] Park W, Seo H, Kim J et al. In-depth correlation analysis between tear glucose and blood glucose using a wireless smart contact lens. Nat Commun 2024; 15: 2828. 10.1038/s41467-024-47123-9 38565532 PMC10987615

[13] Keum DH, Kim S-K, Koo J et al. Wireless smart contact lens for diabetic diagnosis and therapy. Sci Adv 2020; 6: eaba3252. 10.1126/sciadv.aba3252 32426469 PMC7182412

[14] Rasmussen R, Matsumoto A, Dahlstrup SM et al. A segregated cortical stream for retinal direction selectivity. Nat Commun 2020; 11: 831. 10.1038/s41467-020-14643-z 32047156 PMC7012930

[15] Hu Y, Wu T, Guo H et al. Perovskite-based smart eyeglasses as noncontact human–computer interaction. Adv Mater 2025: 2412329. 10.1002/adma.202412329 39821283

[16] Song J-H, Van De Groep J, Kim SJ et al. Non-local metasurfaces for spectrally decoupled wavefront manipulation and eye tracking. Nat Nanotechnol 2021; 16: 1224–30. 10.1038/s41565-021-00967-4 34594006

[17] Cai S, Venugopalan S, Seaver K et al. Using large language models to accelerate communication for eye gaze typing users with ALS. Nat Commun 2024; 15: 9449. 10.1038/s41467-024-53873-3 39487163 PMC11530652

[18] Shi Y, Yang P, Lei R et al. Eye tracking and eye expression decoding based on transparent, flexible and ultra-persistent electrostatic interface. Nat Commun 2023; 14: 3315. 10.1038/s41467-023-39068-2 37286541 PMC10247702

[19] Xu J, Li X, Chang H et al. Electrooculography and tactile perception collaborative interface for 3D human–machine interaction. ACS Nano 2022; 16: 6687–99. 10.1021/acsnano.2c01310 35385249

[20] Wang T, Wang M, Wang J et al. A chemically mediated artificial neuron. Nat Electron 2022; 5: 586–95. 10.1038/s41928-022-00803-0

[21] Cai H, Ao Z, Tian C et al. Brain organoid reservoir computing for artificial intelligence. Nat Electron 2023; 6: 1032–9. 10.1038/s41928-023-01069-w

[22] Pu X, Guo H, Chen J et al. Eye motion triggered self-powered mechnosensational communication system using triboelectric nanogenerator. Sci Adv 2017; 3: e1700694. 10.1126/sciadv.1700694 28782029 PMC5533541

[23] Yao G, Mo X, Liu S et al. Snowflake-inspired and blink-driven flexible piezoelectric contact lenses for effective corneal injury repair. Nat Commun 2023; 14: 3604. 10.1038/s41467-023-39315-6 37330515 PMC10276863

[24] Liu W, Du Z, Duan Z et al. Neuroprosthetic contact lens enabled sensorimotor system for point-of-care monitoring and feedback of intraocular pressure. Nat Commun 2024; 15: 5635. 10.1038/s41467-024-49907-5 38965218 PMC11224243

[25] Zhang S, Chhetry A, Zahed MA et al. On-skin ultrathin and stretchable multifunctional sensor for smart healthcare wearables. npj Flex Electron 2022; 6: 11. 10.1038/s41528-022-00140-4

[26] Ma R, Zhang X, Zhuo J et al. Self-supporting, binder-free, and flexible Ti_3_C_2_T*_x_* MXene-based supercapacitor electrode with improved electrochemical performance. ACS Nano 2022; 16: 9713–27. 10.1021/acsnano.2c03351 35584058

[27] Yan M, Liu S, Liu Y et al. Flexible PVDF-TrFE nanocomposites with Ag-decorated BCZT heterostructures for piezoelectric nanogenerator applications. ACS Appl Mater Interfaces 2022; 14: 53261–73. 36379056 10.1021/acsami.2c15581

[28] Guo M, Guo C, Han J et al. Toroidal polar topology in strained ferroelectric polymer. Science 2021; 371: 1050–6. 10.1126/science.abc4727 33674493

[29] Qin J, Yin LJ, Hao YN et al. Flexible and stretchable capacitive sensors with different microstructures. Adv Mater 2021; 33: 2008267. 10.1002/adma.202008267 34240474

[30] Boutry CM, Negre M, Jorda M et al. A hierarchically patterned, bioinspired e-skin able to detect the direction of applied pressure for robotics. Sci Robot 2018; 3: eaau6914. 10.1126/scirobotics.aau6914 33141713

[31] Hou X, Zhang K, Lai X et al. Brilliant colorful daytime radiative cooling coating mimicking scarab beetle. Matter 2025; 8: 101898. 10.1016/j.matt.2024.10.016

[32] Zhang J, Kim K, Kim HJ et al. Smart soft contact lenses for continuous 24-hour monitoring of intraocular pressure in glaucoma care. Nat Commun 2022; 13: 5518. 10.1038/s41467-022-33254-4 36127347 PMC9489713

[33] Kang S, Lee J, Lee S et al. Highly sensitive pressure sensor based on bioinspired porous structure for real-time tactile sensing. Adv Elect Mater 2016; 2: 1600356. 10.1002/aelm.201600356

[34] Kouhani MHM, Wu J, Tavakoli A et al. Wireless, passive strain sensor in a doughnut-shaped contact lens for continuous non-invasive self-monitoring of intraocular pressure. Lab Chip 2020; 20: 332–42. 10.1039/C9LC00735K 31825423

[35] Sun S, Wang J, Zhang M et al. MEMS ultrasonic transducers for safe, low-power and portable eye-blinking monitoring. Microsyst Nanoeng 2022; 8: 63. 10.1038/s41378-022-00396-w 35711674 PMC9192761

