# Supplementary material for: Encoding of blink information via wireless contact lens for eye–machine interaction
Source: Natl Sci Rev. 2025 Aug 19;12(10):nwaf338. doi: 10.1093/nsr/nwaf338 (PMC12448796; doi:10.1093/nsr/nwaf338)
Supplement: nwaf338_Supplemental_Files [file nwaf338_supplemental_files.zip › Manuscript-SI-final.docx]

**RESEARCH ARTICLE**

**INFORMATION SCIENCE**

**Encoding of blink information via wireless contact lens for eye-machine interaction**

Haiqing Liu^1^, Weijia Liu^1^, Zhijian Du^1^, Lifeng Wu^1^, Minyan Chen^1^, Zhiyi Gao^2^*, Kai Jiang^3^, La Li^1^*, Zhiyong Fan^4^* and Guozhen Shen^1^*

^1^School of Integrated Circuits and Electronics, Beijing Institute of Technology, Beijing 100081, China.

^2^CAS Key Laboratory of Magnetic Materials and Devices, Ningbo Institute of Materials Technology and Engineering, Chinese Academy of Sciences, Ningbo 315201, P. R.

^3^China^.^Faculty of Hepato-Pancreato-Biliary Surgery, Chinese PLA General Hospital, Institute of Hepatobiliary Surgery of Chinese PLA, Key Laboratory of Digital Hepatobiliary Surgery, Beijing, 100853 P. R. China.

^4^Department of Electronic & Computer Engineering, The Hong Kong University of Science and Technology, Kowloon, Hong Kong SAR, China

***Corresponding author.** E-mail: lali@bit.edu.cn, eezfan@ust.hk, [gzshen@bit.edu.cn](mailto:gzshen@bit.edu.cn)

**MATERIALS AND METHODS**

**1. Device fabrication**

**1.1 Microstructured dielectric film**

Initially, the DMF solution of P(VDF-TrFE) was prepared and spin-coated on the sapphire microstructured template at a predetermined spin-coating speed and duration, followed by heating at 80 °C. Due to the hydrophobic nature of P(VDF-TrFE), the microstructured film could be effortlessly stripped from the template using water. This film was subsequently utilized as the dielectric layer of the mechanosensitive capacitor.

**1.2 Mechanosensitive capacitor**

After preparing the mask plate by laser direct writing, the mechanosensitive capacitor was fabricated using the mask plate by uniformly spraying Ti_3_C_2_T*_x_* MXene onto both sides of the plasma-treated dielectric film. When spraying, the film was placed on a heating surface at 60-70 °C, and the distance between the spray gun and the film was 20 mm.

**1.3 Electrical connections and device packaging**

The PDMS solution (PDMS resin and hardener in a mass ratio of 7:1) was then spin-coated onto a glass plate as a substrate and dried at 90 °C for a designated period. Following this, a highly dense aluminum film was deposited onto the PDMS layer and laser etched to form a pattern of the inductive coil, comprising three turns with a pitch of 200 μm. Subsequently, the mechanosensitive capacitor was electrically connected to the inductive coil, encapsulated with an additional layer of PDMS film, and hot-pressed overnight within a copper contact lens mold to impart curvature to the device, thereby producing the final EMI lens.

**2. Cellular experiments**

After thawing the HUVEC cells, they were cultured in a complete medium containing 10% fetal bovine serum at 37 °C with 5% CO_2_ saturated humidity. When the cell density reached 80%, the cells were passaged at a ratio of 1:3 to facilitate further expansion of the culture. HUVEC cells that were in the logarithmic growth phase and exhibited optimal growth conditions were then seeded into 6-well plates at a density of 5×10^4^ cells per well and divided into groups based on the duration of culture. Following the designated culture period, the cells were allocated into two groups: one group received the addition of CCK-8 reagent, and the absorbance at 450 nm was measured using an enzyme labeling apparatus; the other group was treated with Calcein-AM/PI live/dead cell double-staining reagent and subsequently imaged using a confocal microscope.

**3. Blinking eye experiments**

For experiments on the isolated porcine eyes, frozen porcine eyes were first thawed at room temperature for 2 h. After excising unnecessary components, including the outer eyelids, saline was injected into the anterior chamber of the porcine eye with a syringe until the eyeball returned to its normal shape. The EMI lens was cleaned with alcohol and placed in the direct center of the cornea, while the reading coil connected to the VNA was placed approximately 4 mm above the eye. The third eyelid of the porcine eye was clamped with tweezers and slid over the EMI lens every certain time (about 2 s) to simulate the blinking of the porcine eye, and the change of the resonant frequency was recorded during this process. When testing on the eyeball model (which allows for blinking, squinting, eye rotation, etc.), Vaseline was applied for lubrication after placing the EMI lens on the 3D-printed made eyeball due to the high friction between the model components. In experiments involving live rabbits, the rabbit was immobilized using a head clip, and then the reading coil was positioned about 4 mm in front of the rabbit's eyes. The EMI lens was sterilized and immersed in contact lens care solution for 5 minutes before being placed in the rabbit's eye without causing any apparent discomfort. A laser pointer was employed to stimulate the rabbit to close its eye, thereby eliciting a blinking response, and the resonance frequency of the device was recorded during this process. Three three-month-old New Zealand white rabbits were used simultaneously for all experiments involving rabbits in this work (this experiment and the one in Note S5.3) to exclude the interference of individual differences. Animal experiments were conducted in accordance with the guidelines of the Supervision and Administration of Animal Experiments Committee of the Ministry of Health of China and approved by the Beijing Municipal Administration of Laboratory Animals (Approval No. JLHK-20241111-01).

The Institutional Review Board (IRB) of Beihang University approved the contact lens wear experimental protocol (BM20250005), and all experimental procedures were performed with the informed consent of the participants. In addition, we obtained all participants’ consent to use and publish information that identifies individuals, including indirect identifiers. Participants agreed to include images of their eyes with the EMI lenses. At the end of our study, participants were compensated for their time. Before wearing the EMI lens, this contact lens was rinsed using a commercial contact lens care solution (Bausch & Lomb Eye Care Products Ltd). After wearing the EMI lens, participants waited for 30 minutes, during which time they engaged in normal activities. Then, the physiological state of the participants' eyes was observed and their wearing feelings were recorded.

**4. Image processing and control algorithms**

To achieve real-time control of the motion state of the drone, it is imperative to accurately detect the state of the eye and convert it into control commands in real time. Firstly, the image recognition technique is utilized to obtain the resonant frequency information from the S_11_ image in the VNA software in real time. In this process, the computer screen intercepts the screen every 0.1 seconds and then filters the image in the RGB color gamut according to the color difference to find the outline of the S_11_ curve. From the S_11_ curve, the coordinate of the lowest point is found, and the real-time resonance frequency of the EMI lens is obtained from its horizontal coordinate. The resonance frequency information is continuously recorded, and when the resonance frequency changes and the amount of change exceeds the set threshold, the duration of the change is detected, which is used to determine whether the eye behavior is conscious blinking. After determining that the eyes are consciously blinking, the number of eye blinks or the length of eye closure within a certain period of time is recorded, thereby determining the content of the control instructions for the drone. After sending commands to the remote control of the drone through the serial port, the drone performs the corresponding actions.

**5. Drone flight experiments**

In the eye model assisted control drone experimental session, a controlled rectangular flight space was first divided. Subsequently, the EMI lens was placed on the eye model, while the reading coil and VNA were connected. After starting the drone to fly to a preset altitude, the eye model was controlled to perform regular blinking to observe whether the drone was flying in accordance with the preset trajectory. The drone was positioned at a height of approximately 1 meter above the ground to reduce potential interference with flight stability from ground airflow bounce. The optical flow sensor used in the experiment (which monitors the flight attitude of the drone) was extremely sensitive to light, and therefore it was important to provide the experimental environment with sufficient light for the sensor to function properly when testing at night. In the experimental session of rabbit-assisted control of the drone, a two-week animal acclimatization breeding was first conducted to ensure that the experimental subjects were in the best physiological state. Prior to the experiment, the EMI lenses that had been strictly sterilized and treated with a professional care solution were worn on the rabbit's eyes, while an external reading coil was placed next to its eyes. After starting the control program, the operator manually and precisely controlled the duration of the rabbit's eye closure, and simultaneously monitored and recorded the flight status of the drone in real time. At the end of the experiment, the rabbits were subjected to a comprehensive physiological and behavioral assessment, focusing on the presence of eye abnormalities, normal locomotor activity, and maintenance of natural feeding behavior.

**Note** **S1. Materials source and synthesis**

**1.1 Materials source**

TiC, Al and Ti powders were supplied by Alfa Aesar. Hydrochloric acid (HCl), hydrofluoric acid (HF) and anhydrous lithium chloride (LiC) were purchased from Alladin Reagents, Inc. N, N-Dimethylformamide (DMF) was procured from Innochem. P(VDF-TrFE) (80-20% mol) was offered by Beijing Apsilon Technology Co. Polydimethylsiloxane (PDMS, Dow Corning DC184) was sourced from Biofount. No further purification was required for the reagents used in the experiment.

**1.2 Ti_3_AlC_2_ MAX**

Ti_3_AlC_2_ MAX was produced by high temperature annealing of TiC, Al and Ti powders. The powders were mechanically pre-activated by ball milling for 20 hours at a zirconia ball to powder weight ratio of 2:1. The milled powder was placed in a tube furnace and the samples were heated at high temperature for a period of time under a continuous flow of argon gas. After cooling, Ti_3_AlC_2_ MAX powders were obtained by grinding porous briquettes and sieving through a sieve.

**1.3 Mono-layered Ti_3_C_2_T*_x_* MXene**

Mono-layered Ti_3_C_2_T*_x_* MXene was fabricated by HF etching [1]. Firstly, a certain mass of Ti_3_AlC_2_ powder was added in small amounts to a solution containing a concentration of deionized water, hydrochloric acid, and hydrofluoric acid, and then stirred for one full day at 38 °C in an oil bath. The solution is removed from the oil bath and the steps of centrifugation and washing of the precipitate with deionized water are repeated until the pH of the supernatant is greater than 5. Up to this point, the multi-layered Ti_3_C_2_T*_x_* MXene is produced. Subsequently, the precipitate is diluted with deionized water, followed by the addition of a certain mass of anhydrous lithium chloride and stirred at room temperature for 12 hours. After washing 3 times to remove excess impurities, the centrifugation and shaking steps were repeated to obtain pure mono-layered Ti_3_C_2_T*_x_* MXene.

**Supplementary Note 2: Material characterization.**

**2.1 Characterization methods**

The surface morphology and elemental composition of Ti_3_C_2_T*_x_* MXene were studied using field emission transmission electron microscopy (FETEM) equipped with an energy spectrometer (EDS) and a high-resolution transmission electron microscope (HRTEM). The surface and thickness information of the Ti_3_C_2_T*_x_* MXene nanosheets was further obtained using atomic force microscopy (AFM, Dimension lcon). The lattice structure of Ti_3_AlC_2_ MAX and Ti_3_C_2_T*_x_* MXene was determined by X-ray diffraction (XRD, Bruker D8 Advance). The elemental composition and intermolecular interactions of MXene were investigated using Raman spectroscopy (Renishaw inVia).

**2.2 Characterization of Ti_3_AlC_2_ and Ti_3_C_2_T*_x_***

The X-ray diffraction experiments reveal the lattice structure of Ti_3_AlC_2_ and Ti_3_C_2_T*_x_*. Ti_3_AlC_2_ MAX has multiple diffraction peaks, with the strongest Al diffraction peak at about 39°, pointing to the (104) lattice plane (Fig. S2, bottom). After etching, the diffraction peaks corresponding to the (002) lattice face of multi-layered Ti_3_C_2_T*_x_* MXene become broader and the offset angle becomes smaller compared to that of Ti_3_AlC_2_ MAX (Fig. S2, middle). The high-angle diffraction peaks in the top curve of Fig. S2 almost disappear, and there is only a distinct characteristic peak (002) at about 7°, which is due to the formation of mono-layered Ti_3_C_2_T*_x_* MXene after Li ion intercalation [2]. Fig. S3a clearly demonstrates a 2D Ti_3_C_2_T_x_ MXene nanosheet with a size of more than 3 μm, while the dark-field image (Fig. S3b) reveals the typical hexagonal lattice structure of MXene [3]. The EDS spectrum (Fig. S4a) depicts the elemental composition of the Ti_3_C_2_T_x_ MXene, and it can be seen that the elements Ti and C are the most abundant [4]. Fig. S4b-d exhibit the elemental distribution of aluminum and titanium elements in MXene: Al is essentially etched away by acid etching, while Ti is uniformly distributed on the 2D MXene nanosheet. The lattice spacing is known to be 0.236 nm, corresponding to the 002 crystalline surface of Ti_3_C_2_T*_x_* MXene, from the HRTEM image (Fig. S5) [5]. In addition, a single crystal region of MXene can be clearly identified, which illustrates regularly oriented lattice stripes. The thickness of the MXene nanosheet is approximately 1.707 nm, as illustrated in the AFM image (Fig. S6) [6]. Furthermore, the Raman spectrum shown in Fig. S7 is divided into four regions. Starting from the resonance peak coupled to the plasma peak (laser wavelength: 785 nm), it is followed by flake region consisting of the E_g_ (Ti, C, O) and A_1g_ (Ti, C, O) modes, T*_x_* region (220-470 cm^-1^) indicating the vibration of the surface groups of titanium atoms, and C region (580-730 cm^-1^) indicating the vibration of the surface groups of carbon atoms [7].

**Supplementary Note 3: Finite element analysis.**

**3.1 Mechanical simulation**

Firstly, a three-dimensional model of the eye was created, which consisted of two parts, the sclera and the cornea, where the cornea encompassed approximately the anterior 1/6 of the ocular surface, with a corneal thickness of 1 mm, and the sclera wrapped around the ocular surface in approximately the posterior 5/6 of the ocular surface. Thereafter, two circular capacitors with different structures were designed. To realize the maximum change of capacitance, the capacitors were attached to the corneoscleral junction position of the eye, which underwent covariant deformation with the eye. The capacitor consisted of two electrode layers and one dielectric layer, which was categorized into microstructured and unstructured dielectrics, and was subjected to mechanical and electrical simulations under equivalent conditions, respectively. When the capacitor with microstructured dielectric deformed, the air domain between the electrodes also deformed, hence the need to introduce a dynamic mesh to maintain an accurate description of the changes in the dielectric layer.

After applying uniform pressure on the inner wall of the eye, stress-strain simulations were performed on the whole model, and the simulation results are shown in Fig S9. To specifically study the capacitance change, a radial cross-section was chosen and the deformation of the capacitor and the cornea over this cross-section was investigated under different pressure conditions (from 0 to 50 mmHg with an interval of 10 mmHg), yielding the strain distributions shown in Fig. S10. It can be observed that under identical pressure conditions, capacitors with microstructured dielectrics exhibit greater radial deformation, which corresponds to larger capacitance variations.

**3.2 Electrical simulation**

In the global definition, a terminal voltage of 1 V was applied to the electrode on the side close to the cornea, and a ground voltage of 0 V was applied to the electrode on the side away from the cornea, while the changes in the electrical parameters were subsequently monitored at different pressures. Fig. S11 presents the potential distribution in the radial section, and it can be seen that the initial potential distribution of the microstructured capacitor and the unstructured capacitor are similar, with the 0-1 V potential surface distributed along the radial direction. However, as the pressure increases (from 0 to 50 mmHg with an interval of 10 mmHg), the 0-1 V potential surface gradually deviates from the radial surface, which is due to the fact that the capacitor is subjected to both tangential and radial forces when pressure is applied to the inner wall of the eye. In this process, the capacitor with microstructured dielectric deforms more under the intraocular pressure, and therefore the 0-1 V potential surface deviates more from the initial potential surface. In addition, by simulating the electric field distribution and charge accumulation between capacitors during IOP changes, the change in capacitance was evaluated by using the equation $C=Q/U$,which in turn evaluated the sensitivity of microstructured and unstructured capacitors to the pressure response.

**Supplementary Note 4: Calculation.**


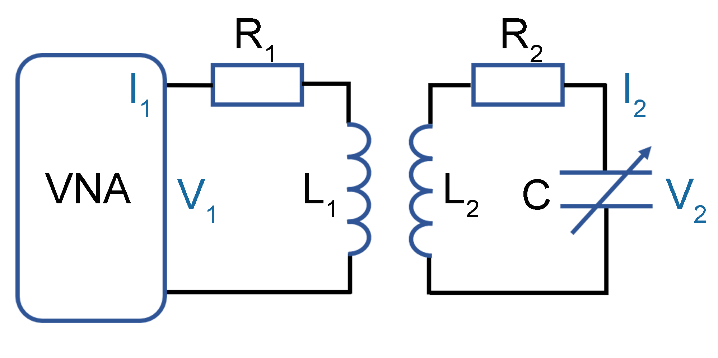


**Figure Note 1**

In the sensor circuit system, the capacitance of the sensitive capacitor $C$ is:

$$\begin{aligned} C=\frac{\varepsilon S}{d}\#\left( S1 \right) \end{aligned}$$

When the sensitive capacitor is pressurized ($\Delta P$), the dielectric layer produces a displacement $\Delta d$.

$$\begin{aligned} \Delta d=\delta\cdot\Delta P\#\left( S2 \right) \end{aligned}$$

Thus the capacitance $C$ of the sensitive capacitor can be expressed as:

$$\begin{aligned} C=\frac{\varepsilon S}{d-\Delta d}=\frac{\varepsilon S}{d-\delta\cdot\Delta P}\#\left( S3 \right) \end{aligned}$$

At resonance $f_{2}$, the impedances of capacitor and inductor cancel each other, minimizing the impedance of the entire sensor circuit system. The resonant frequency and quality factor of the sensor circuit system are shown below:

$$\begin{aligned} f_{2}=\frac{1}{2\pi\sqrt{L_{2}C}}=\frac{\sqrt{d-\delta\cdot\Delta P}}{2\pi\sqrt{L_{2}\varepsilon S}}\#\left( S4 \right) \end{aligned}$$

$$\begin{aligned} Q=\frac{1}{R_{2}}\sqrt{\frac{L_{2}}{C}}\#\left( S5 \right) \end{aligned}$$

Applying Kirchhoff's law to the VNA detection circuit and the sensor circuit in the Laplace domain, the following results are obtained:

$$\begin{aligned} V_{1}=I_{1}R_{1}+j\omega L_{1}I_{1}+j\omega MI_{2}\#\left( S6 \right) \end{aligned}$$

$$\begin{aligned} V_{2}=I_{2}R_{2}+j\omega L_{2}I_{2}+j\omega MI_{1}\#\left( S7 \right) \end{aligned}$$

Where $V_{1}$, $V_{2}$ denote the voltage across the VNA and the voltage across the capacitor, and$I_{1}$, $I_{2}$ denote the current in the VNA detection circuit and the current in the sensor circuit. M is the inductive coupling between the sensing coil and the reading coil, which satisfies $M=k\sqrt{L_{1}L_{2}}$. The value of the coupling coefficient k is in the range of $[-1, 1]$, which indicates the maximum coupling when $k=1$ and no coupling when $k=0$.

The voltage-current relationship across the sensitive capacitor can be expressed as:

$$\begin{aligned} \frac{V_{2}}{I_{2}}=\frac{1}{j\omega C_{2}}\#\left( S8 \right) \end{aligned}$$

The equivalent input impedance Z_1_ can be obtained by associating Eqs. S6, S7 and S8:

$$\begin{aligned} Z_{1}=\frac{V_{1}}{I_{1}}=j2\pi fL_{1}\left( 1+\frac{k^{2}\left( \frac{f}{f_{2}} \right)^{2}}{1+\frac{jf}{f_{2}}Q-\left( \frac{f}{f_{2}} \right)^{2}} \right)\#\left( S9 \right) \end{aligned}$$

$$\begin{aligned} \mathrm{Re}\left( Z_{1} \right)=R_{1}+2\pi fL_{1}k^{2}Q\frac{\frac{f}{f_{2}}}{1+Q^{2}\left( \frac{f}{f_{2}}-\frac{f_{2}}{f} \right)^{2}}\#\left( S10 \right) \end{aligned}$$

If $Q\gg1$, $\mathrm{Re}\left( Z_{1} \right)$ reaches its maximum when $f=f_{2}$. If the modulus of $S_{11}$ is set to $r$, then $Z_{1}$ can be expressed as:

$$\begin{aligned} r=\left| S_{11} \right|\in\left[ 0, 1 \right)\#\left( S11 \right) \end{aligned}$$

$$\begin{aligned} \frac{Z_{1}}{Z_{0}}=\frac{1+S_{11}}{1-S_{11}}=\frac{1+re^{i\theta}}{1-re^{i\theta}}=\frac{1-re^{-i\theta}+re^{i\theta}-r^{2}}{1-re^{-i\theta}+re^{i\theta}-r^{2}}\#\left( S12 \right) \end{aligned}$$

Where $Z_{0}$ is the port impedance of the VNA. The following is obtained by simplifying Eqs. (S12):

$$\begin{aligned} \frac{Z_{1}}{Z_{0}}=\frac{1-r^{2}+2irsin\left( \theta\right)}{1-2rcos\left( \theta\right)+r^{2}}\#\left( S13 \right) \end{aligned}$$

$$\begin{aligned} Re\left( Z_{1} \right)=\frac{1-r^{2}}{1-2rcos\left( \theta\right)+r^{2}}\cdot Z_{0}\#\left( S14 \right) \end{aligned}$$

When $r\to0$,

$$\begin{aligned} Re\left( Z_{1} \right)=\frac{1-r^{2}}{1-2rcos\left( \theta\right)+r^{2}}\to1\#\left( S15 \right) \end{aligned}$$

As $r$ increases, since $1-2rcos\left( \theta\right)+r^{2}\geq\left( 1-r \right)^{2}\geq1-r^{2}$,

$$\begin{aligned} Re\left( Z_{1} \right)=\frac{1-r^{2}}{1-2rcos\left( \theta\right)+r^{2}}\leq1\#\left( S16 \right) \end{aligned}$$

And the smaller $r$, the closer this value is to 1. Therefore, the modulus of $S_{11}$ is smallest when $Re(Z)$ is largest, namely when $f=f_{2}$. As a result, the resonant frequency of the sensor circuit system can be estimated by the frequency at which the modulus of $S_{11}$ is minimized.

**Supplementary Note 5: Cellular experiments and CLS-wearing tests**

**5.1 Cell proliferation assay**

**5.1.1 Cellular resuscitation**

(1) After the HUVEC cells were removed from the liquid nitrogen, they were quickly placed in a water bath at 37 °C and the cryopreservation tube was gently shaken to dissolve the cryopreservation solution;

(2) After lysis, the cells were transferred to a centrifuge tube containing 5 ml of medium, centrifuged to collect the cells (centrifuged at 1000 rpm for 5 minutes at room temperature), and the supernatant was discarded;

(3) The cells were suspended in complete medium containing 10% fetal bovine serum, inoculated into Petri dishes, gently blown and mixed, and incubated at 37 °C with 5% CO_2_ saturated humidity.

**5.1.2 Cell Reproduction**

The cells were passaged when the cell density reached 80%:

(1) After discarding the medium, the cells were washed once with PBS;

(2) 1 ml of trypsin was added to digest the cells and observed under the microscope. After 2 minutes of digestion, the cells were seen to be separated and rounded, indicating that the digestion was complete;

(3) After the trypsin was rapidly washed away, the remaining cells were added to the complete medium and gently blown to make a single cell suspension, passaging at a ratio of 1:3 and expanding the culture at 37 °C with 5% CO_2_ saturation humidity.

**5.1.3 Cell processing**

(1) HUVEC cells in logarithmic growth phase with good growth status were taken and inoculated into cell culture 6-well plates at a density of 5×10^4^ cells/well, and cultured overnight in an incubator at 37 °C with 5% CO_2_ (200 μL of sterile PBS was added to the wells around the cell wells);

(2) Cells were divided into four groups according to the time of co-cultivation with EMI lens: control, one-day, four-day and seven-day groups.

**5.1.4 Cellular CCK8 assay**

(1) After the required cell culture time, 50 μL of CCK-8 was added to each well of the cell suspension and incubated at 37 °C for 1 hour;

(2) The solution was aspirated into the cell culture 96-well plate according to the standard of 100 μL/well;

(3) The absorbance value of each well was determined at 450 nm using an enzyme marker.

**5.2 Live-dead cell staining assay**

The first three steps of the experiment are the same as those described in Note. S5.1.

**5.2.4 Cellular resuscitation**

(1) Cells were digested with trypsin-EDTA, followed by centrifugation to collect the cells (100 rpm, 3 min).

(2) After supernatant removal, the cells were washed 2-3 times with 1× Assay Buffer to adequately remove residual esterase activity;

(3) The suspension of cells was prepared with 1×Assay Buffer at a density of 1×10^5^-1×10^6^ cells/mL;

(4) 100 μL of staining working solution (working solution concentration of 2 μM for calcein-AM and 4.5 μM for PI) was taken and added to 200 μL of cell suspension, mixed well, and incubated at 37 °C for 15 minutes.

(5) The cell suspension was titrated and then confocally photographed.

**5.3 Wearing tests on rabbits**

Three-month-old New Zealand white rabbits (approximately 3 kg) were used to evaluate the biocompatibility of EMI lens. The EMI lens was sterilized and soaked in contact lens buffer for 5 minutes, after which the EMI lens was worn on the cornea of the rabbit's right eye. Before wearing the contact lens and at 1, 2, and 3 days of wear, respectively, the rabbits were photographed to obtain slit-lamp photographs of the ocular surface and white-light photographs of the eyes. A drop of fluorescent dye was placed in the conjunctival sac of the rabbit's eye before the slit lamp photographs were taken [8]. After the rabbits wore the EMI lens for 3 days, the rabbits were humanely euthanized with highly concentrated potassium chloride, and the eyelid and corneal tissues were collected from the rabbits. The collected tissues were used for histopathologic analysis and reporting after paraffin processing, sectioning, and H&E staining. Three New Zealand White rabbits were subjected to this experiment at the same time to exclude the effects of individual differences.

**Supplementary Note 6: Analog IOP testing platform.**

An IOP test platform was constructed to test the response of the EMI lens to pressure, which consisted of several parts, including a bionic eyeball, a pressure regulating system, and a signal testing system (shown in Fig. S17). The bionic eye is composed of a stainless-steel chamber and a bionic cornea, which is filled with saline to simulate intraocular fluid, and the bionic cornea is used to place the EMI lens. The pressure regulation system is made up of a programmable stepper motor, an infusion tube, and an infusion bag, which connects to the stainless-steel chamber and adjusts the height of the infusion bag through the stepper motor for the purpose of controlling the pressure in the chamber. The signal testing system mainly consists of a manometer (Keller LEX1), a reading coil and a vector network analyzer (Nano VNA). The manometer monitors the IOP in real time, while the VNA monitors the S_11_ data of the coil in real time and presents it in the form of images. When the stepper motor drives the infusion bag to move, the pressure of the chamber changes, which leads to the deformation of the bionic cornea, and the EMI lens deforms conformally with the cornea, and the resonance frequency changes.

During testing, the Keller LEX1 manometer was first zero-calibrated before intracameral insertion to establish baseline pressure reference. Subsequent pressure variations were induced through controlled infusion bag height adjustments, with each pressure level systematically correlated to the corresponding EMI lens resonant frequency for calibration purposes, thereby establishing the pressure-frequency response profiles.

**Supplementary Note 7: Effect of relative position of device and reading coil on baseline frequency.**

To validate whether the relative position of the reading coil to the EMI lens would have an effect on the signal detection of the EMI lens, three experimental variables were designed, including vertical distance, horizontal distance and relative angle, and the resonant frequency of the EMI lens was continuously recorded while changing these three variables. In the experiment of varying the vertical distance, the initial distance between the reading coil and the EMI lens was 2 mm. Afterwards, the EMI lens slowly descended with the Z-axis displacement stage and stopped at 7 mm, and the S_11_ image of the VNA was recorded at every 0.5 mm of descent. In the experiment of changing the horizontal distance, the reading coil and the center of the EMI lens were located in the same vertical line at the beginning. Subsequently, the EMI lens moved slowly outward along with the X-axis displacement table to stop at 6mm, and the S_11_ image of the VNA was recorded at every 1mm of movement. In the experiment of changing the relative angle, the reading coil was relatively parallel to the EMI lens at the beginning. After that, the EMI lens was slowly rotated to stop at 50°, while the S_11_ image of the VNA was recorded at every 10° of rotation. The experiments indicate that the resonant frequency of the EMI lens does not change beyond the threshold regardless of the change of the vertical distance (Fig. S18), horizontal distance (Fig. S19), or relative angle (Fig. S20) between the reading coil and the EMI lens, which proves that the relative position of the reading coil and the EMI lens does not have an effect on the signal detection of the EMI lens.

**Supplementary Note 8: Effect of environment on baseline frequency.**

To verify whether the experimental environment affects the performance of the coil, the effects in terms of temperature, humidity, cellular phone signals and light condition were applied to the EMI lens, respectively, and its resonance frequency was continuously detected at the same time. In the temperature test, the EMI lens and the reading coil were placed together in an oven, and the temperature inside the oven was gradually changed (from 20 °C to 70 °C, with S_11_ data output every 10 °C). As shown in Fig. S21, when the temperature is gradually increased from 20 °C to 40 °C, the resonance frequency of EMI lens remains constant; when the temperature is gradually increased from 40 °C to 70 °C, the resonance frequency of EMI lens gradually increases, which is due to the fact that the high temperature makes the contact lenses indicate more obvious deformation. However, since the temperature of the human eye stays around 36-37 °C, the regular use of the EMI lens is not affected. In the humidity test, the EMI lens and the reading coil were placed in a closed chamber, and the relative humidity of the environment was increased from 30% to 80% by ultrasonic atomization of water. As shown in Fig. S22, with the resonance frequency of the device basically remaining unchanged. In the cell phone signal test, the cell phone is placed next to the EMI lens, the test begins after a period of time the phone receives a call and continues to talk for about 1 minute. As shown in Fig. S23, the resonant frequency of the device did not change significantly during the phone call. To evaluate the impact of light conditions on the EMI lens, we conducted relevant experiments (Fig. S24). The resonance frequency of the EMI lens was continuously monitored throughout the process. Initially, measurements were taken under normal lighting conditions, followed by exposing the EMI lens to intense illumination for one minute. The experimental results demonstrate that the baseline resonance frequency of the EMI lens showed no obvious variation before and after intense illumination.

**Supplementary Note 9: Conscious blink detection and drone control.**

**9.1 Conscious blink detection**

To prevent eye dryness and fatigue, a person performs 15-20 unconscious blinks per minute [9]. When wearing the EMI lens, unconscious blinking also causes a decrease in resonance frequency, but because unconscious blinking puts less pressure on the cornea and takes less time, it can be programmed to distinguish between unconscious blinking and conscious blinking (Fig. S31). The EMI lens is worn on both eyes. When the resonance frequency significantly decreases, the base value of this frequency is used to determine whether it pertains to the left or right eye, followed by the detection of the magnitude and duration of the frequency drop. If the magnitude and duration of the drop are greater than the set threshold, it is determined that the human eye produces conscious blinking behavior, which can be converted into subsequent computer control commands; if the magnitude or duration of the drop is less than the set threshold, it is determined that the human eye is blinking unconsciously, which is of no practical significance.

**9.2 Drone control**

As illustrated in Fig. S32, after the control commands are transmitted to the drone's remote controller via the serial port, the remote controller forwards these commands to the drone's communication module using the NRF24L01 transmitter chip. The communication module then relays the signals to the STM32 main controller, which is responsible for controlling the MPU6050 gyroscope (to detect the drone's flight attitude) and the four-channel brushed motor (to provide flight power and control the flight direction). The SPI protocol is implemented between the master controller and the communication module to ensure accurate and timely transmission of control commands to the drone. Meanwhile, the I2C protocol is used between the master controller and the gyroscope, facilitating the connection of multiple slave devices (a six-axis gyroscope). Additionally, the communication rate of the I2C protocol is adequate to meet the data transmission requirements of the MPU6050 gyroscope.


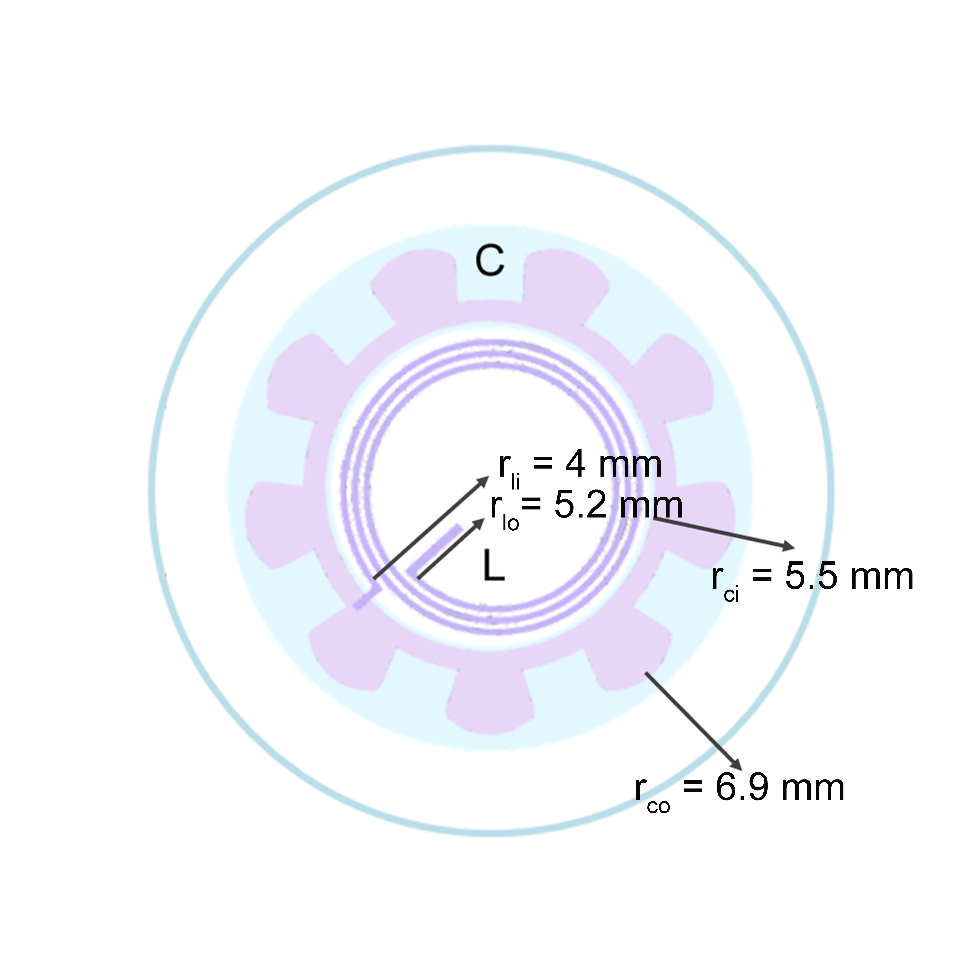


**Figure S1.** Structural and parametric characterization of capacitor and inductor for EMI lens.


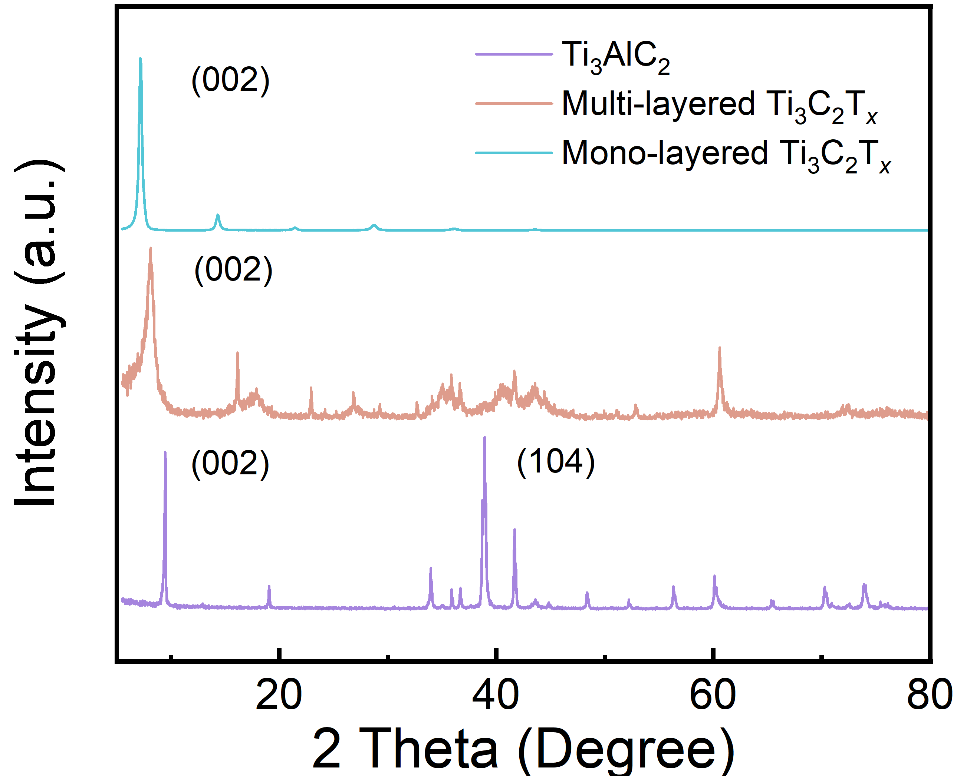


**Figure S2.** XRD pattern of Ti_3_AlC_2_, multi-layered Ti_3_C_2_T*_x_* MXene and mono-layered Ti_3_C_2_T*_x_* MXene.


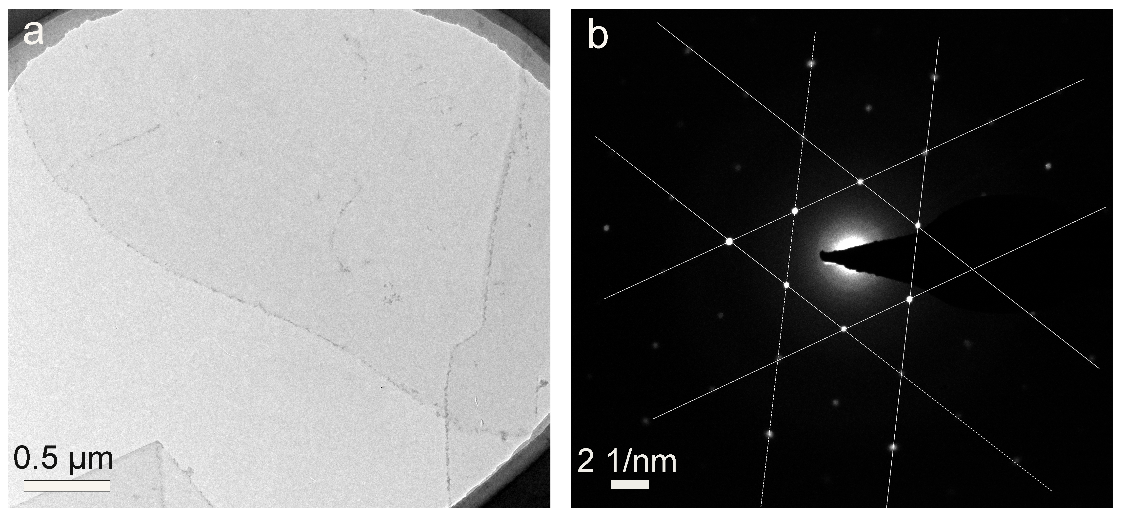


**Figure S3.** TEM image (a) and dark field image (b) of mono-layered Ti_3_C_2_T*_x_* MXene.


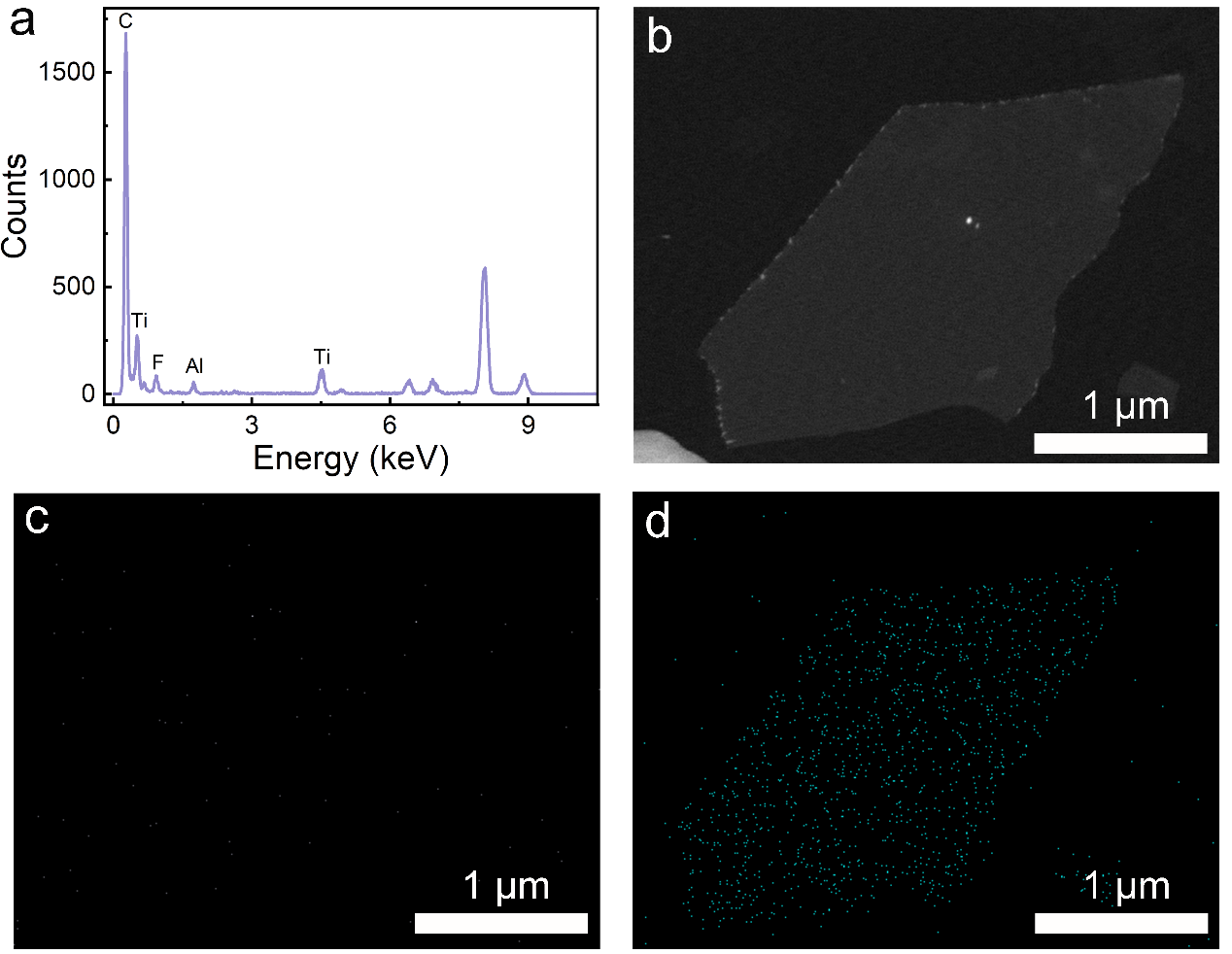


**Figure S4.** EDS characterization of mono-layered Ti_3_C_2_T*_x_* MXene. (a) EDS energy spectrum. (b) TEM view of mono-layered Ti_3_C_2_T*_x_* MXene. Regional distribution of Al (c) and Ti (d).


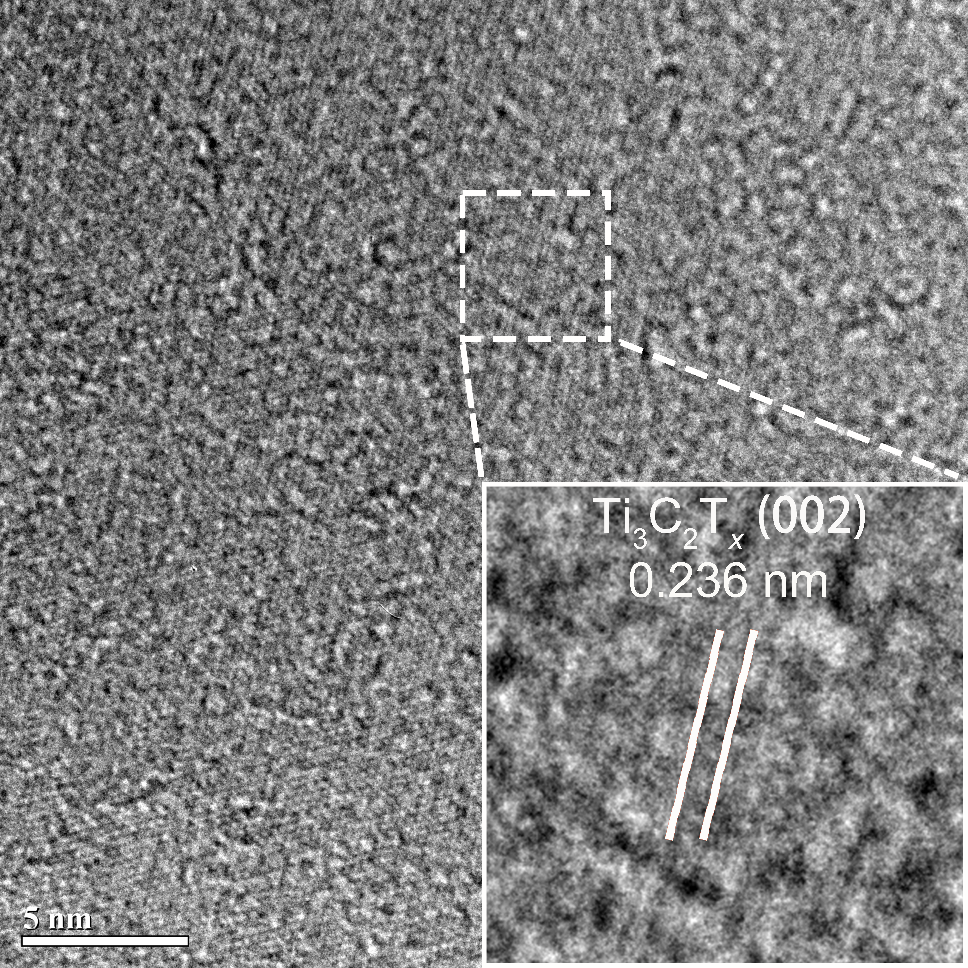


**Figure S5.** HRTEM image of mono-layered Ti_3_C_2_T*_x_* MXene.


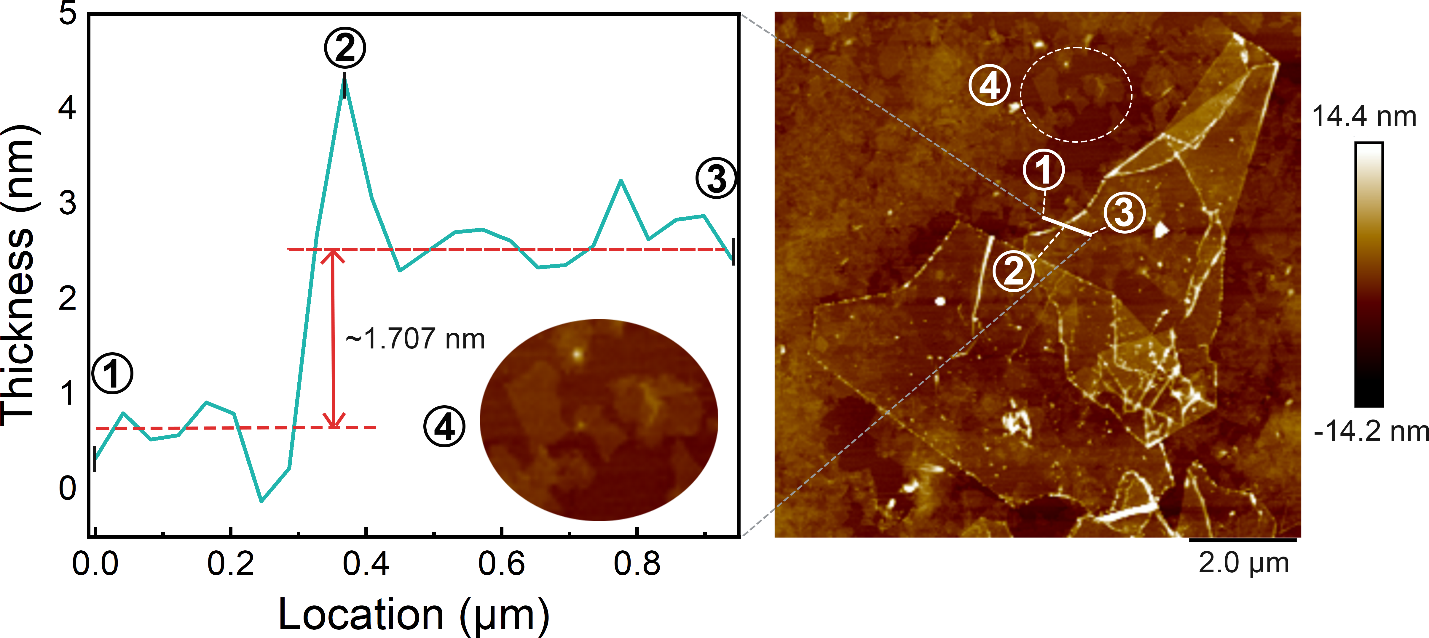


**Figure S6.** Thickness profile (left) and AFM image (right) of mono-layered Ti_3_C_2_T*_x_* MXene.


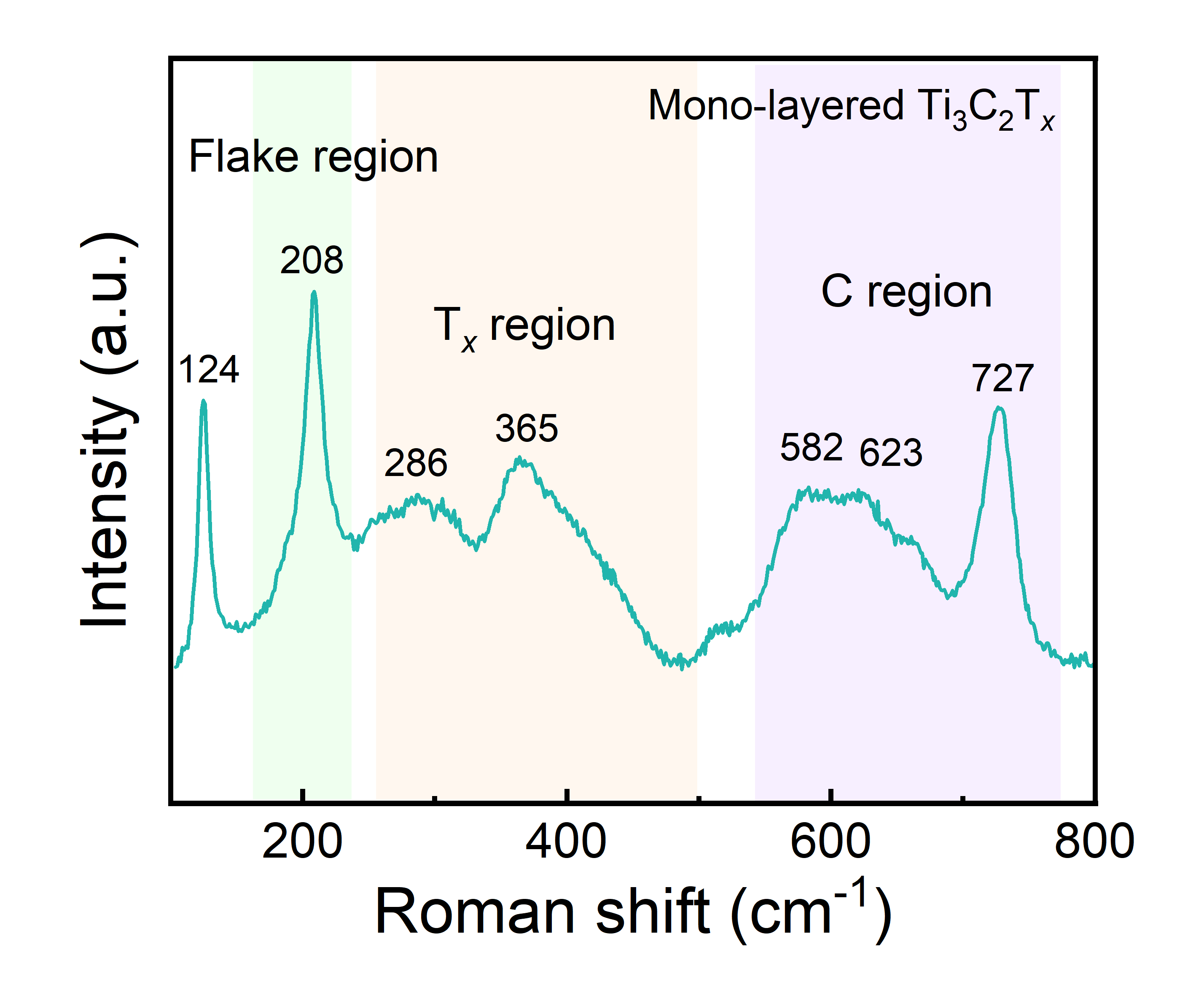


**Figure S7.** Schematic of Raman peak distribution of mono-layered Ti_3_C_2_T*_x_*.


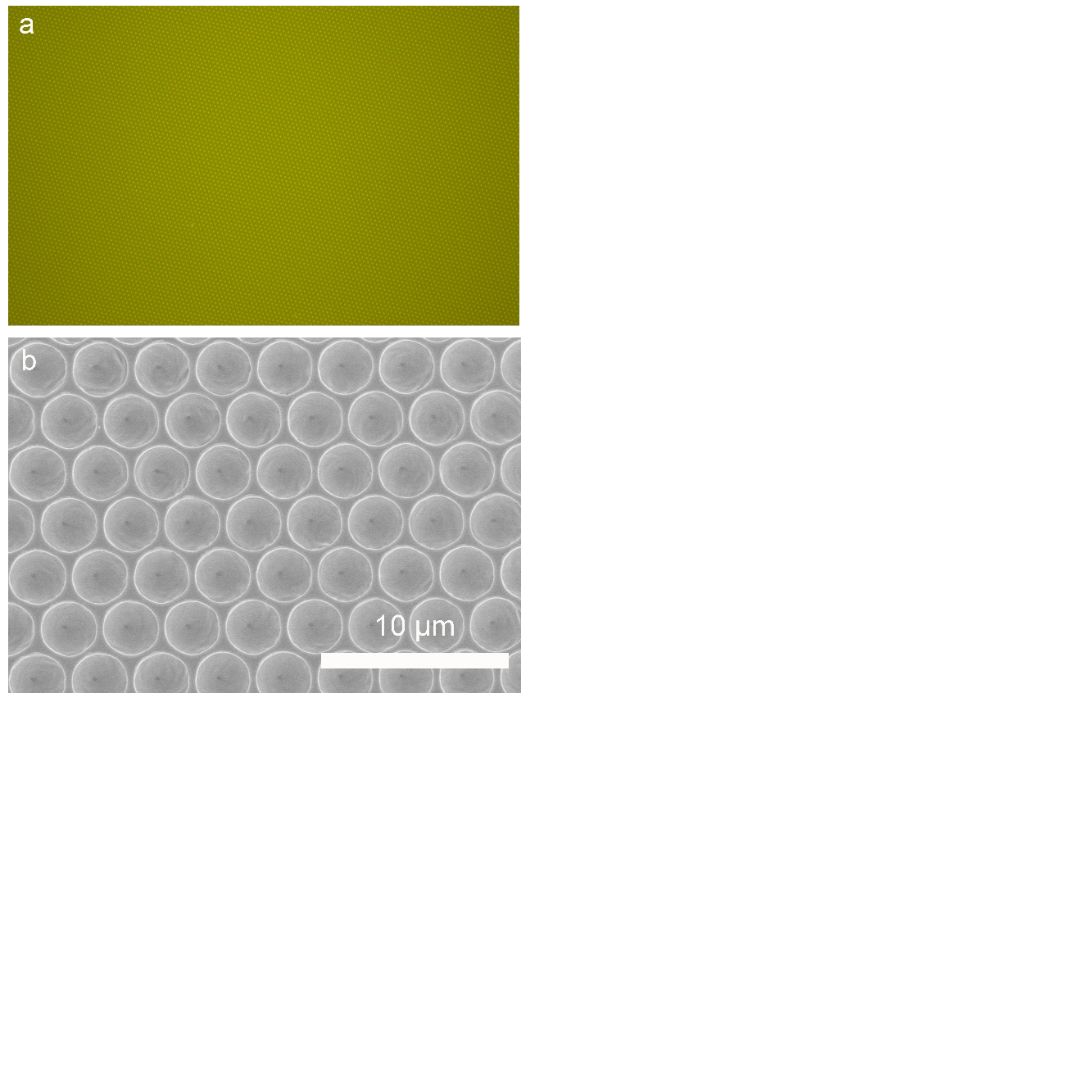


**Figure S8.** Microscopic image (a) and SEM image (b) of microstructured dielectric film.


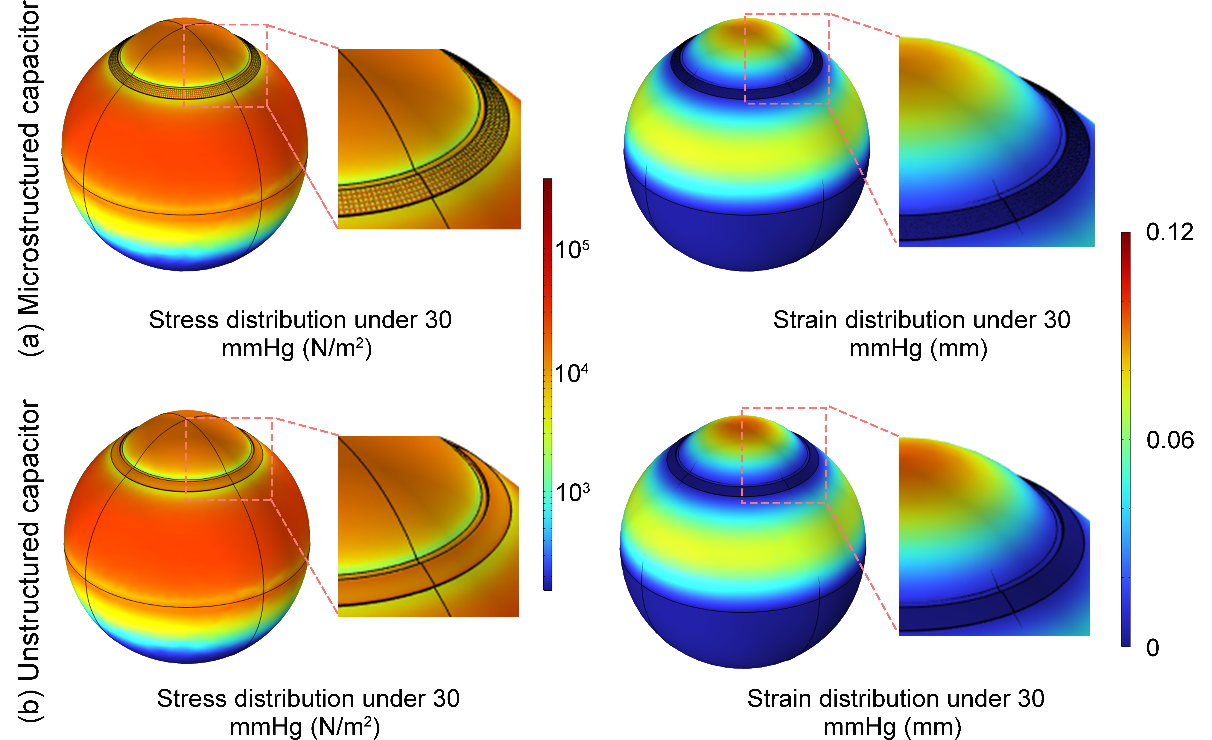


**Figure S9.** COMSOL simulations of stress and strain at 30 mmHg pressure for the eye model wearing (a) microstructured and (b) unstructured capacitors.


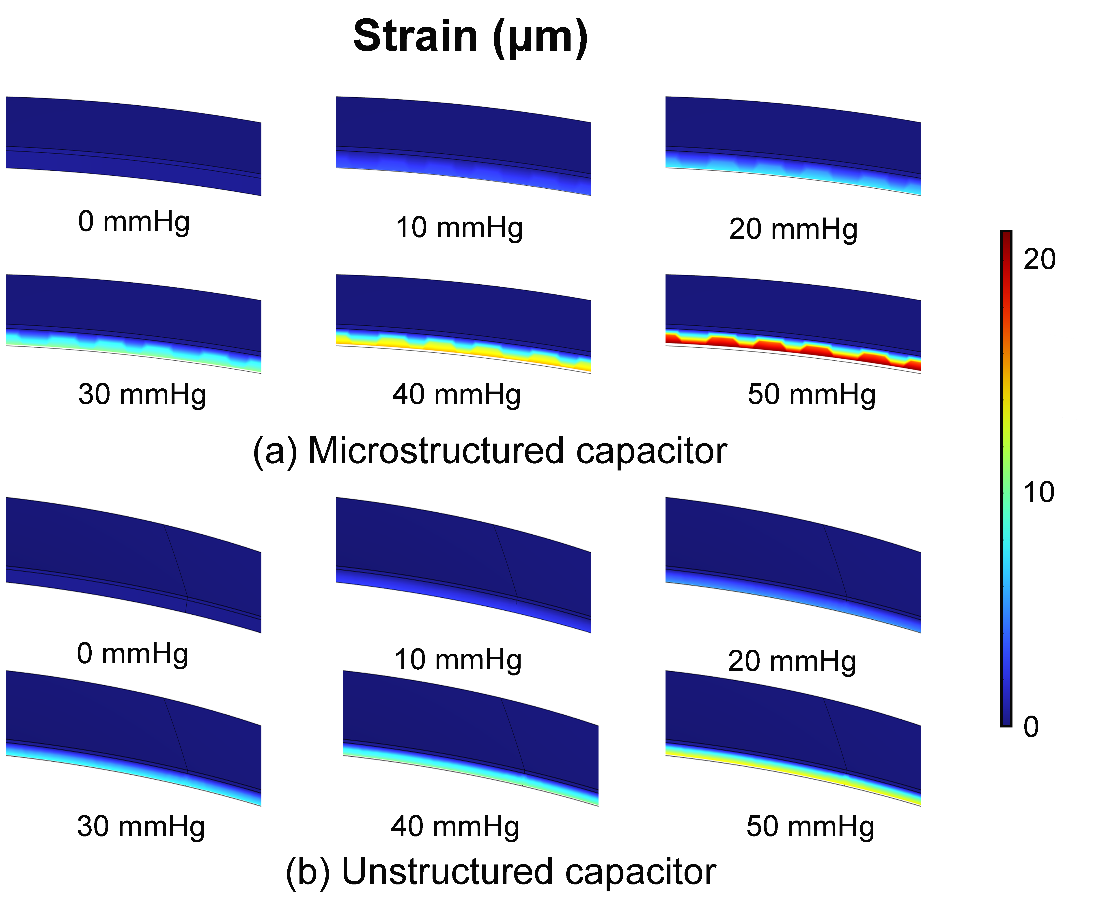


**Figure S10.** COMSOL simulation of strain at different pressures (0-50 mmHg) for microstructured and unstructured capacitor on an eyeball model.


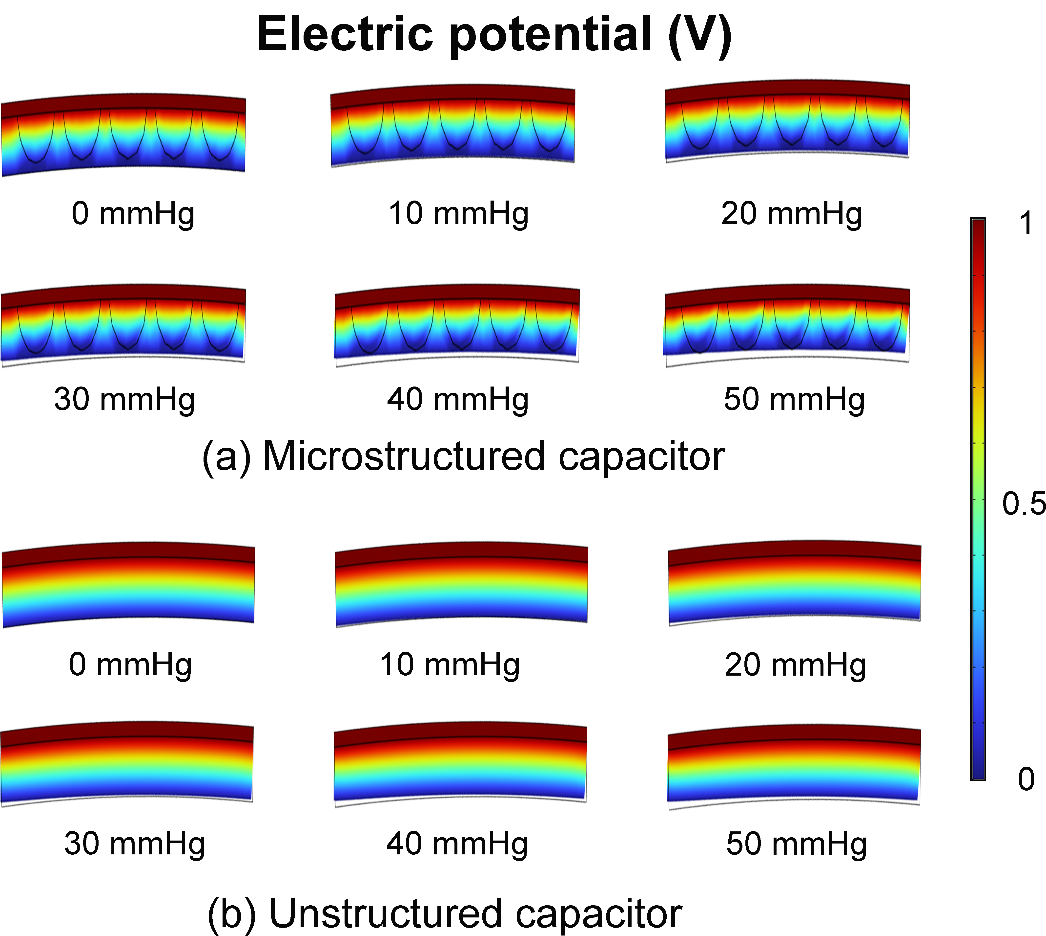


**Figure S11.** COMSOL simulation of electric potential at different pressures (0-50 mmHg) for microstructured and unstructured capacitor on an eyeball model.


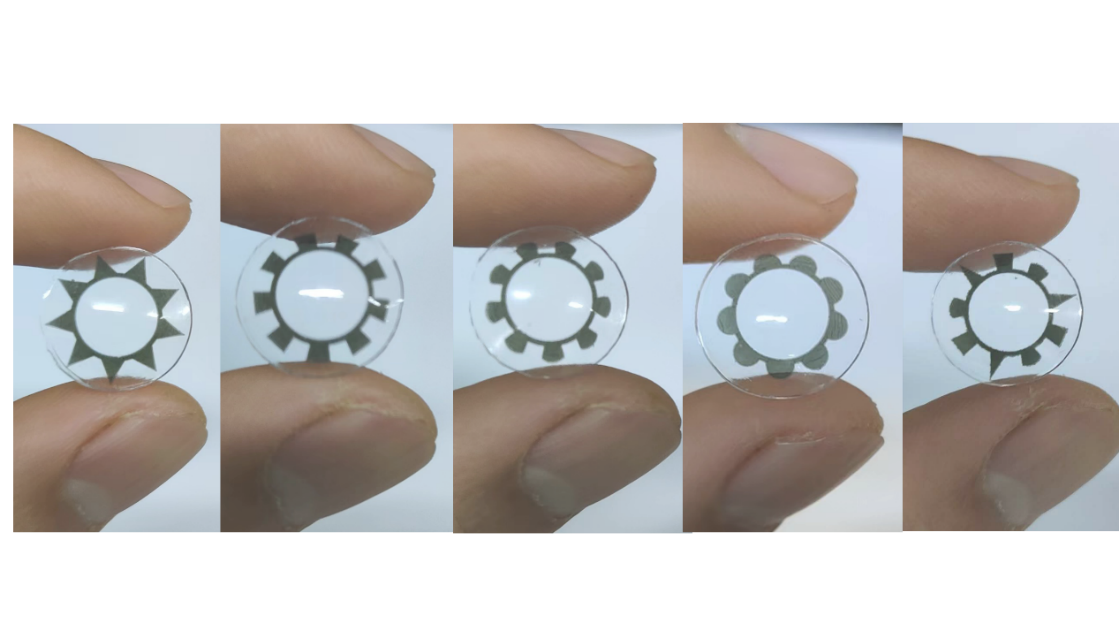


**Figure S12.** Sensitive capacitors with different shapes.


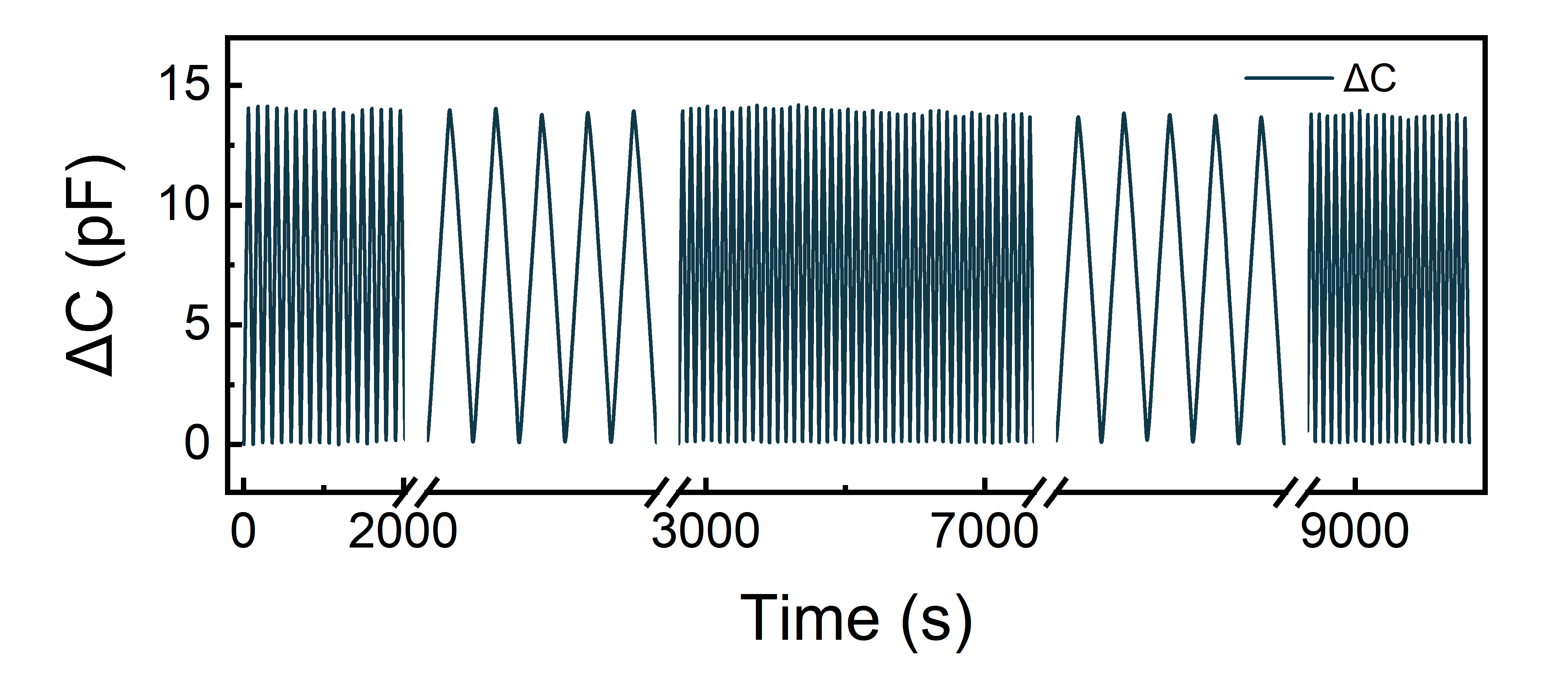


**Figure S13.** Capacitance change of device subjected to prolonged cycling in the pressure range of 0-70 mmHg.


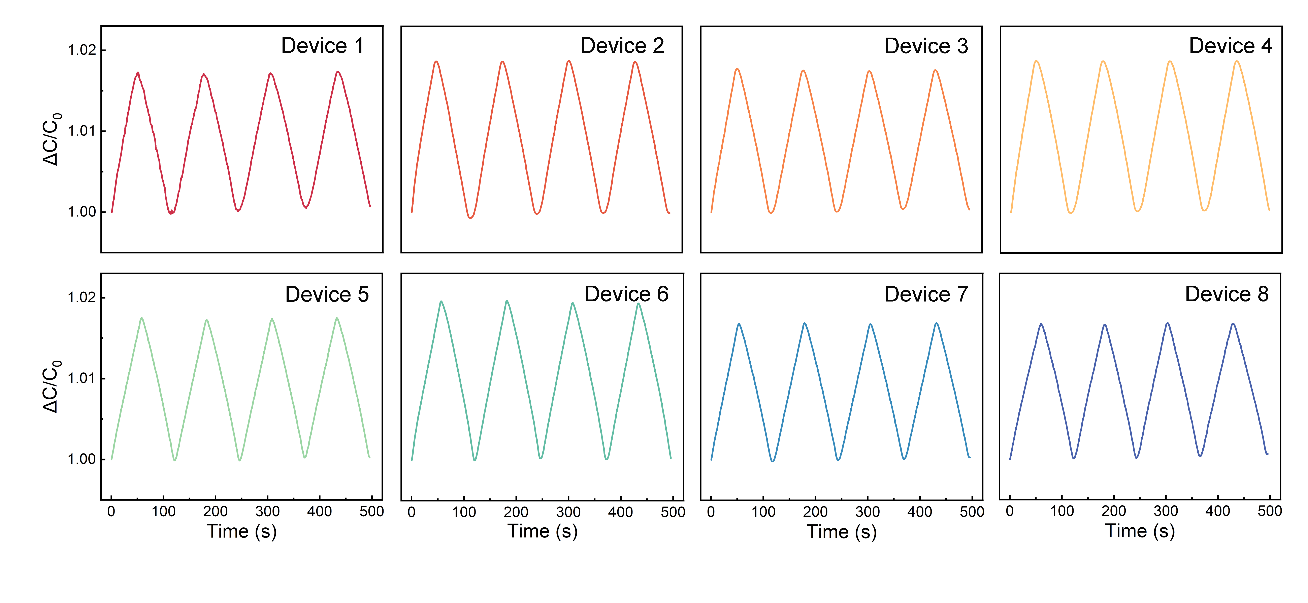


**Figure S14.** Ratio of capacitance to initial capacitance (ΔC/C_0_) for different devices s cycled over a long period of time in the pressure range 0-70 mmHg.


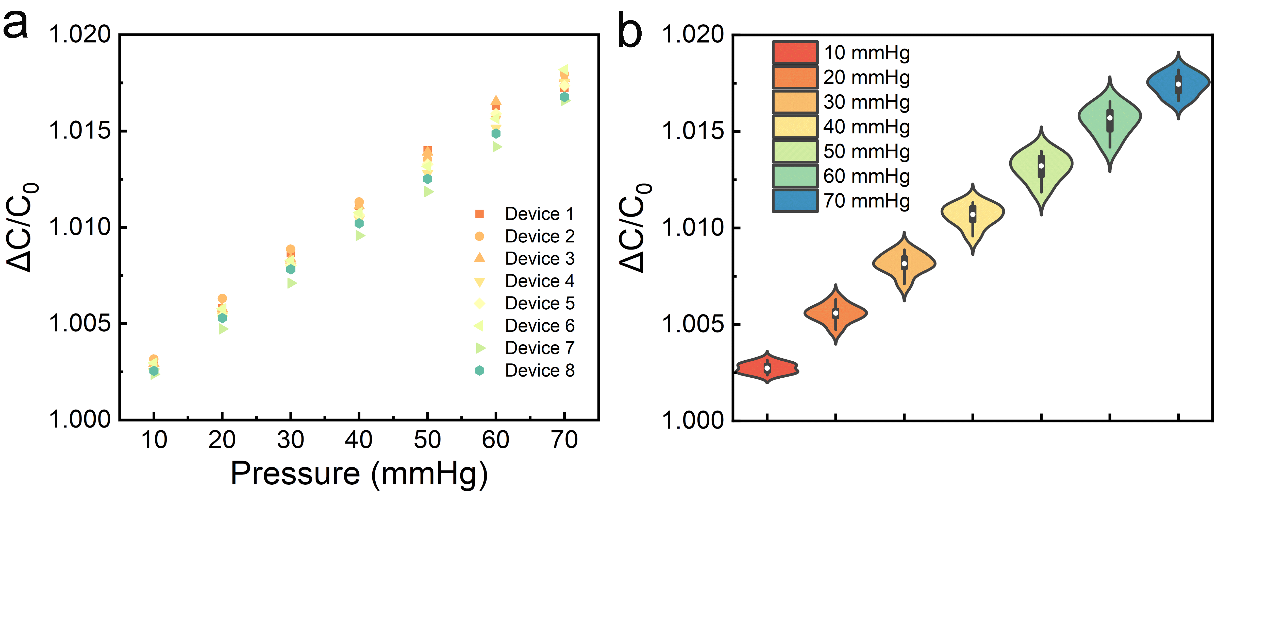


**Figure S15.** Ratio of capacitance to initial capacitance (ΔC/C_0_) (a) and error distribution (b) for different devices at different pressures.


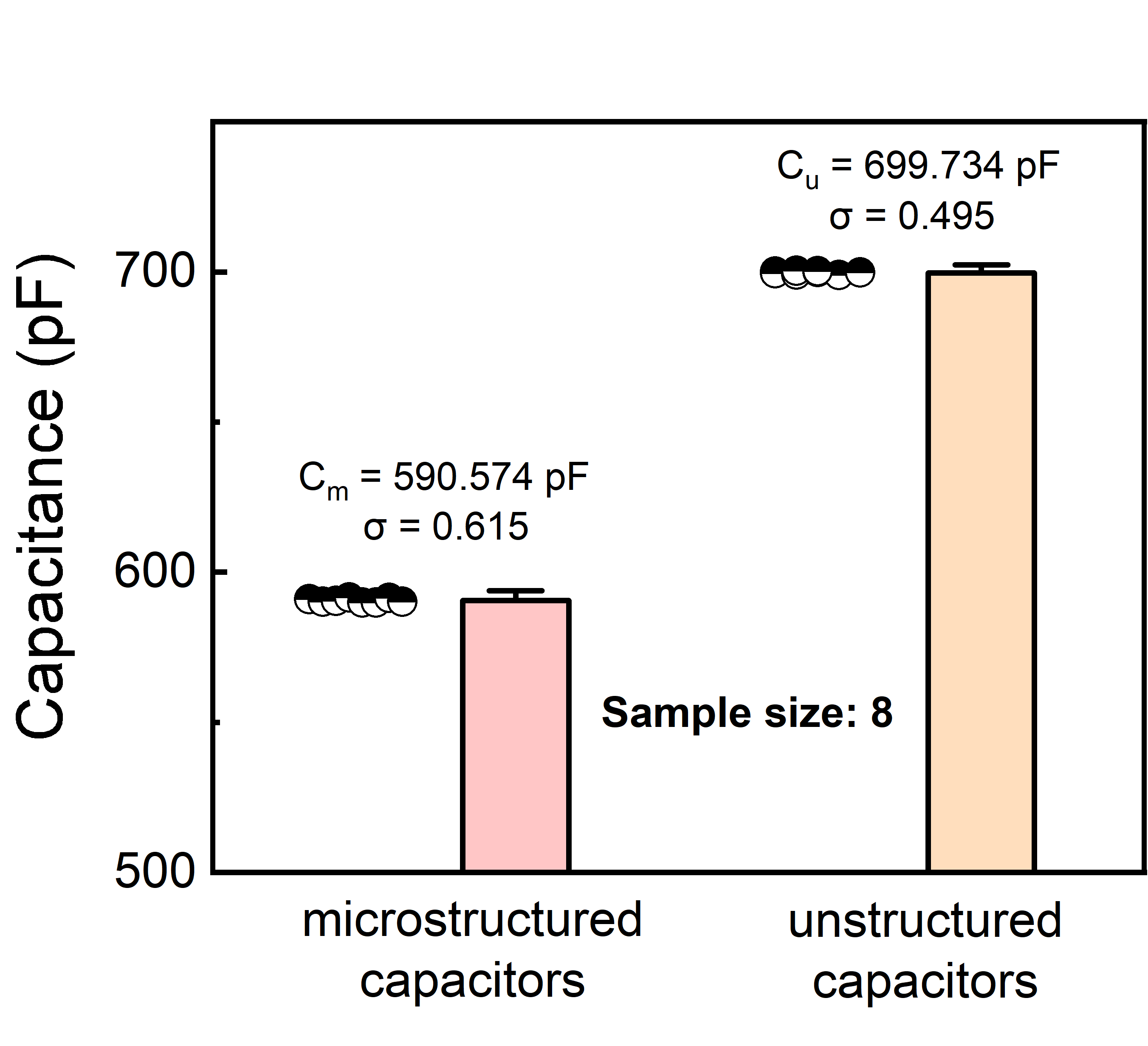


**Figure S16.** Statistical analysis of capacitance values for microstructured and unstructured capacitors.


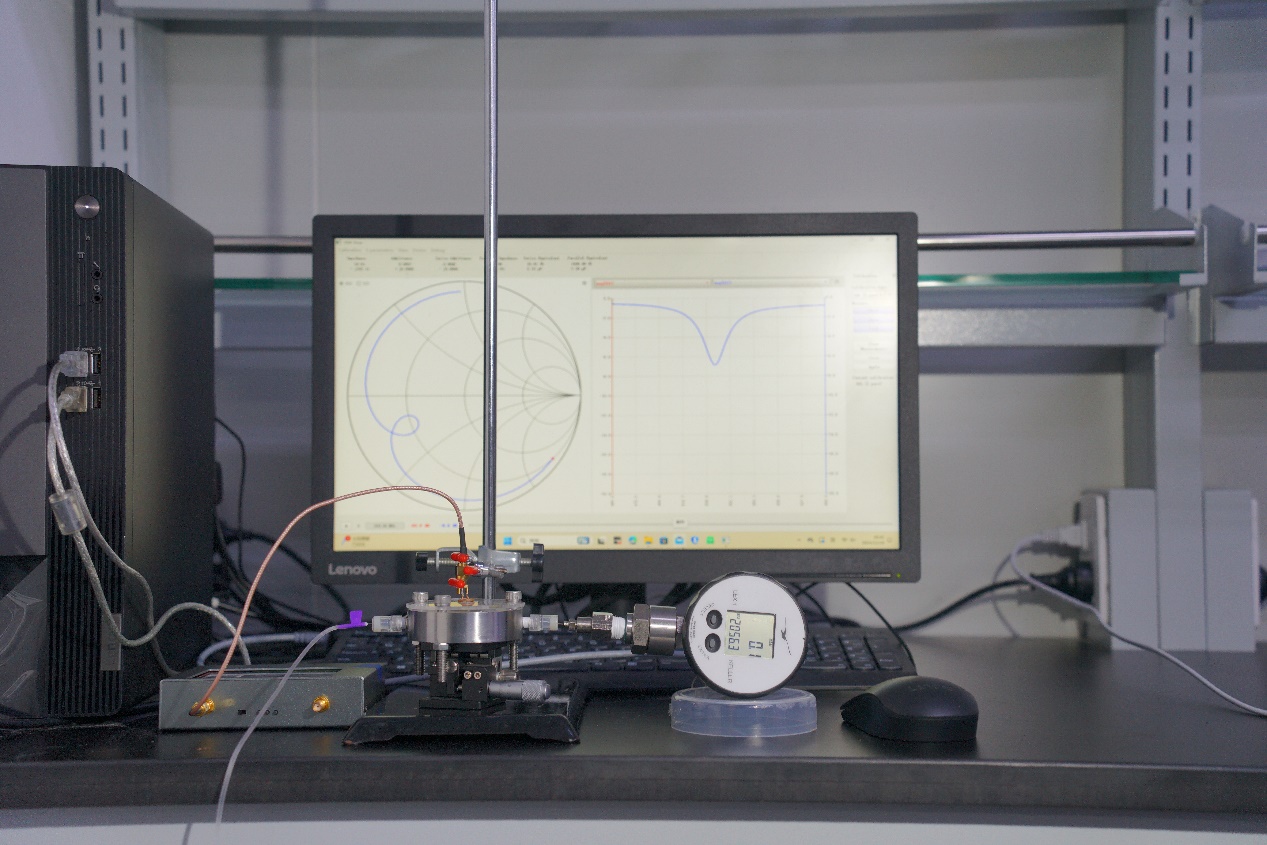


**Figure S17.** Photograph of analog stress test rig including analog eyeball, stepper motor, manometer and VNA.


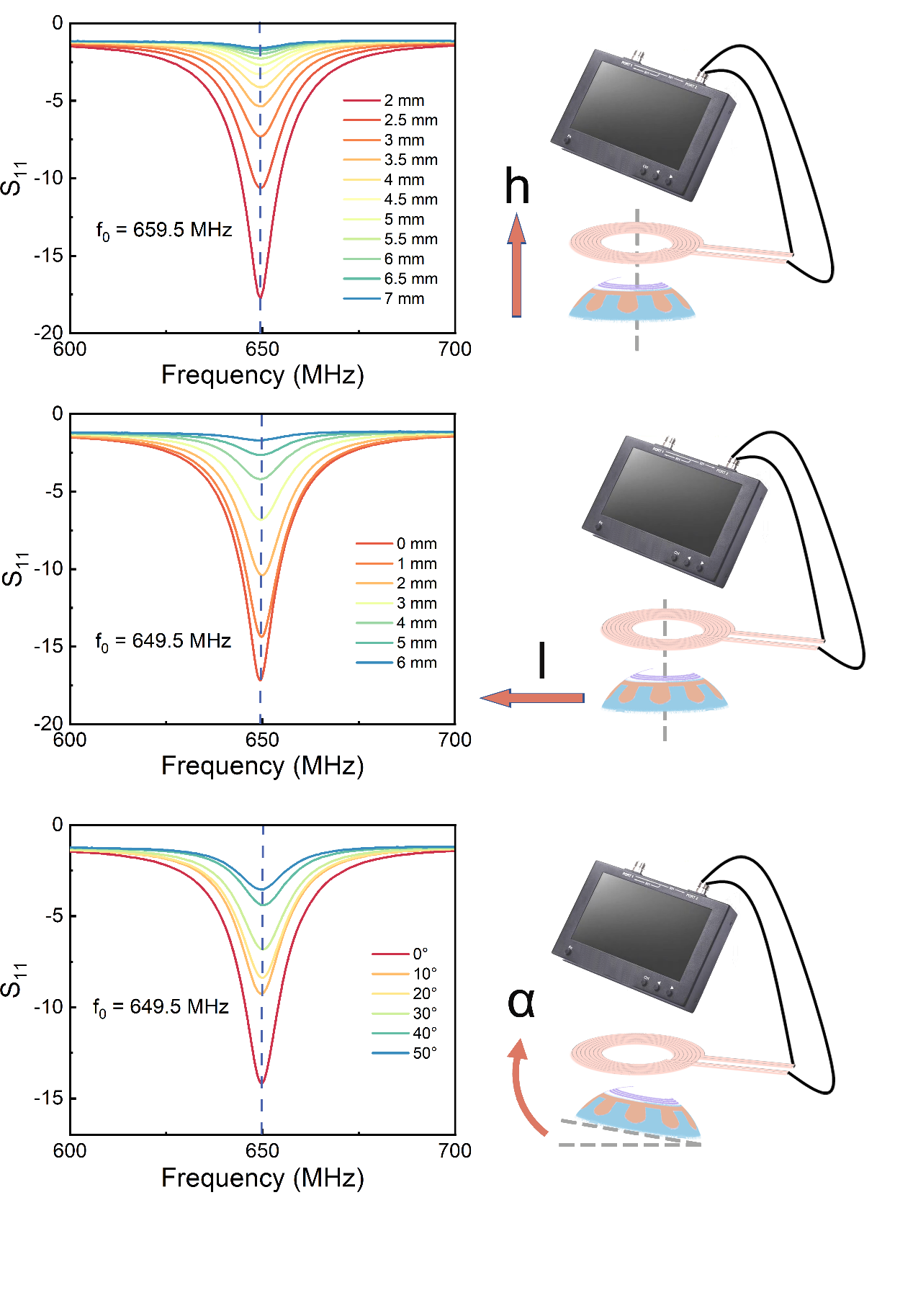


**Figure S18.** Effect of vertical distance between EMI lens and reading coil on baseline frequency.


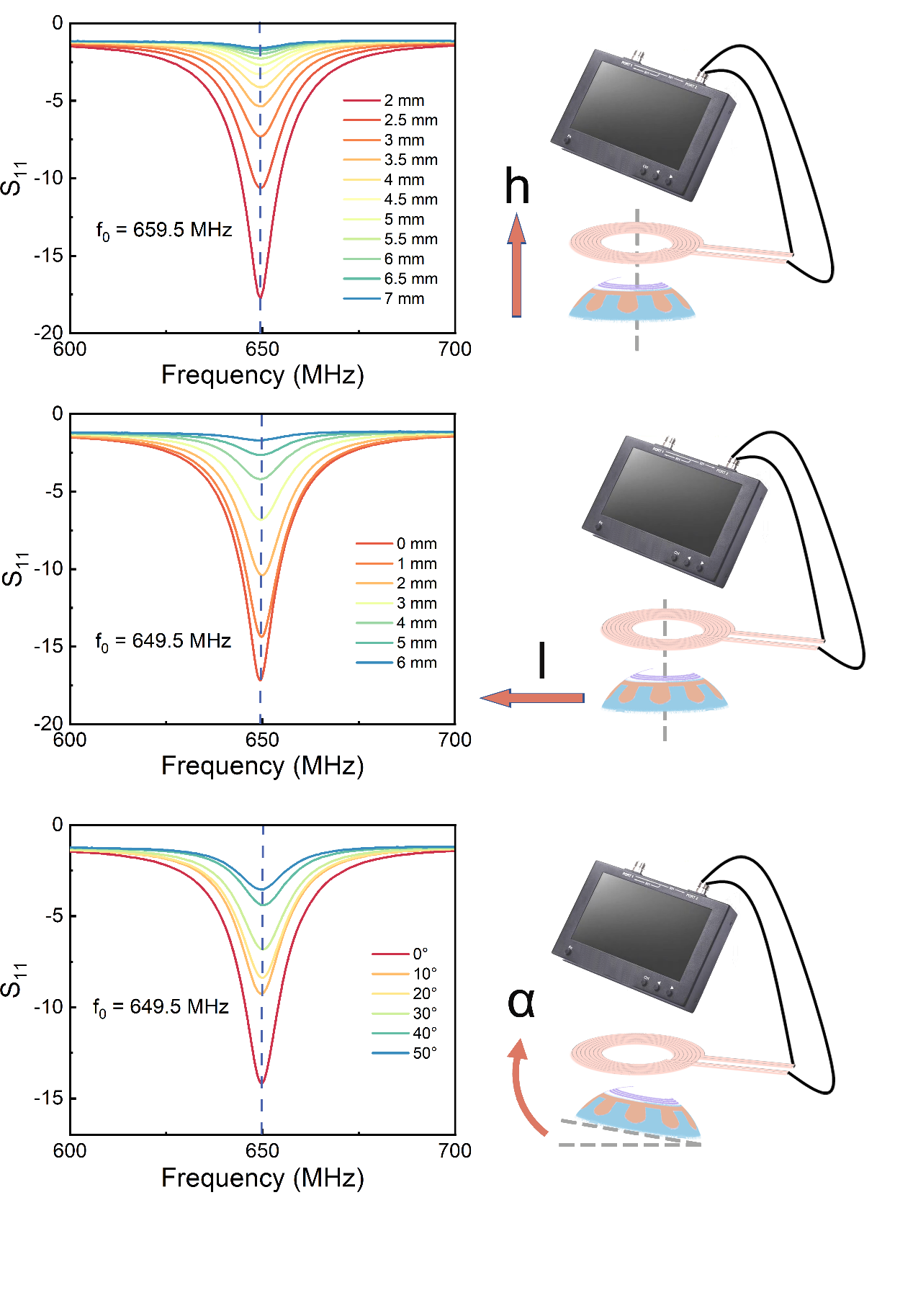


**Figure S19.** Effect of horizontal distance between EMI lens and reading coil on baseline frequency.


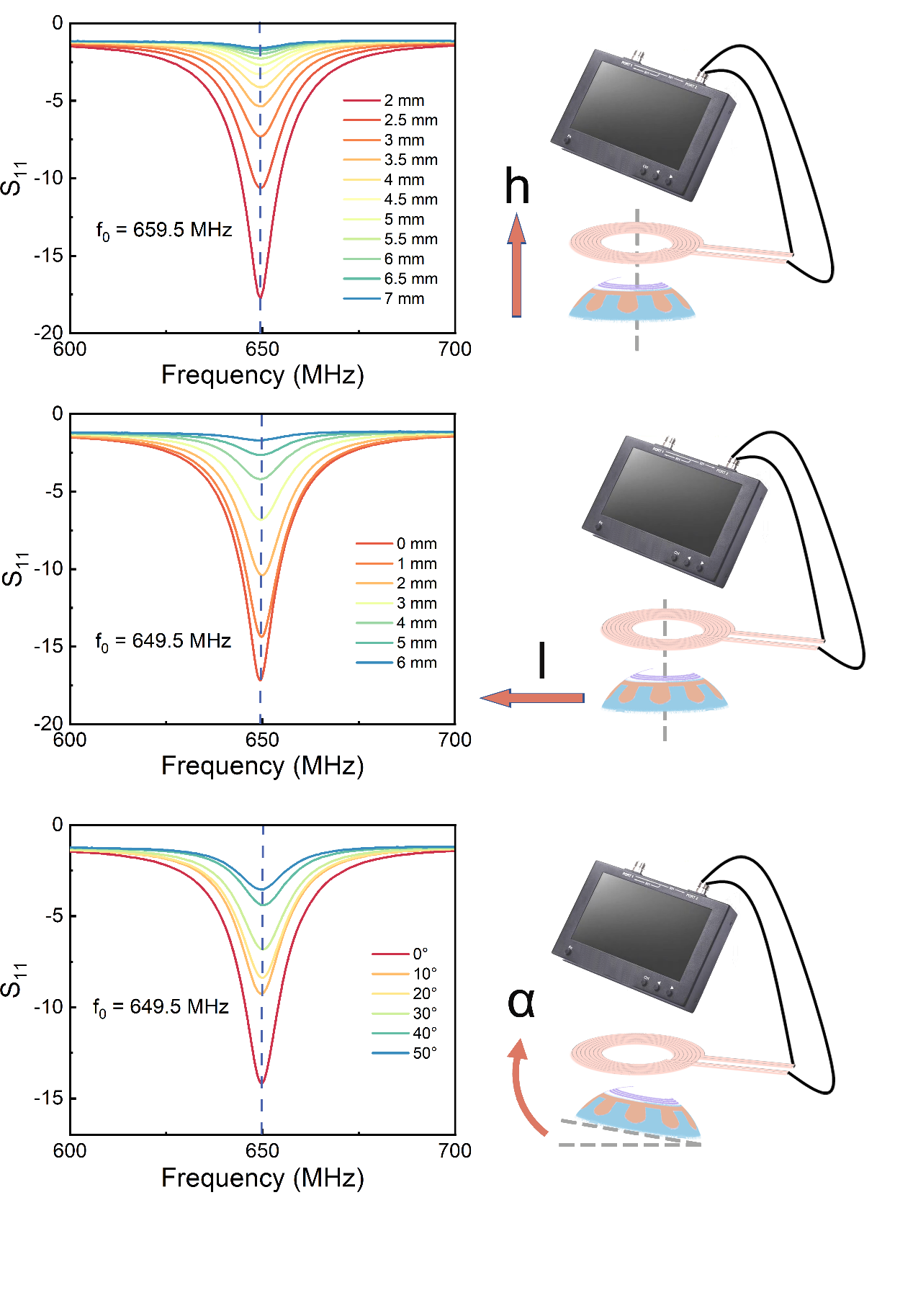


**Figure S20.** Effect of angle between EMI lens and reading coil on baseline frequency.


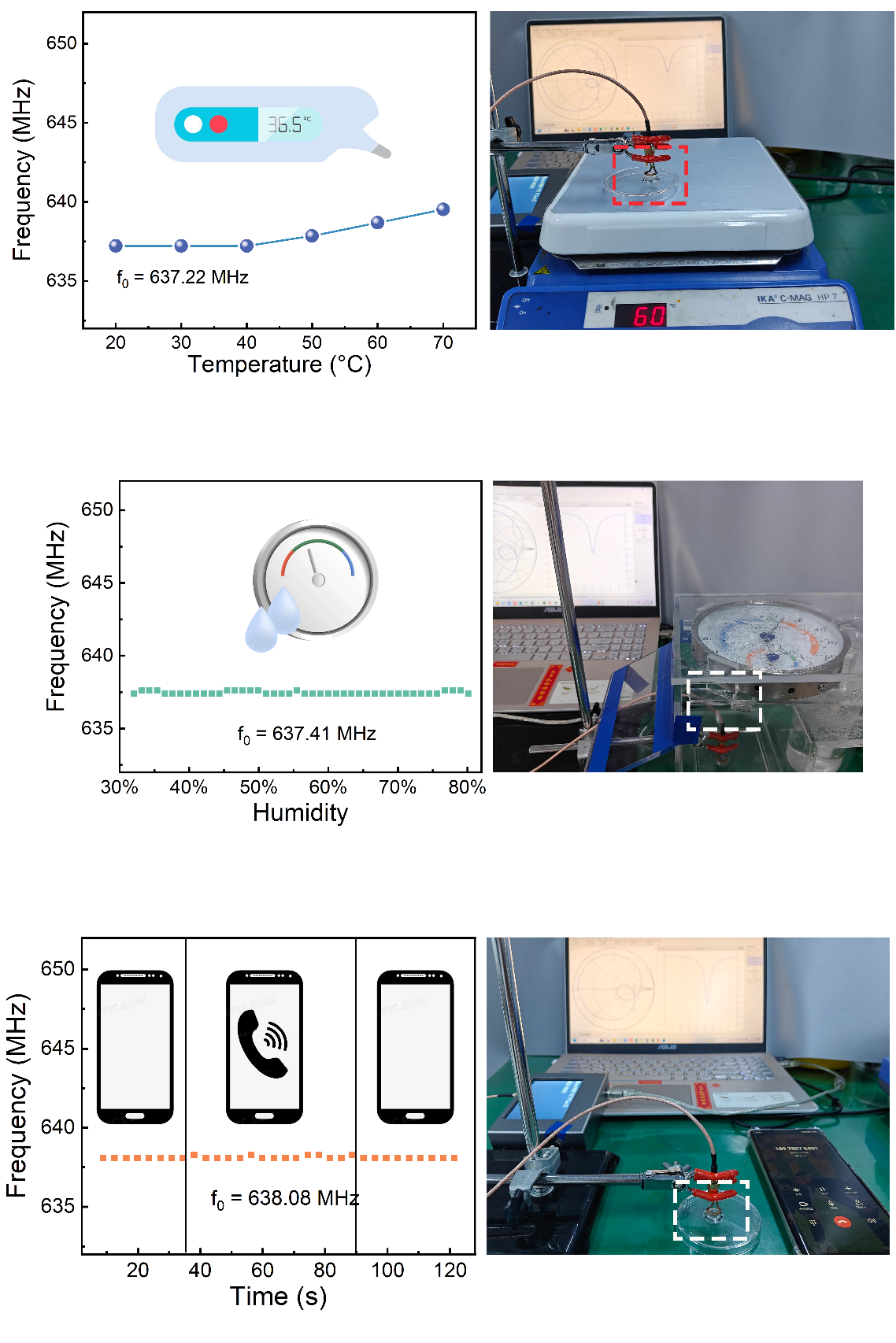


**Figure S21.** Effect of temperature on the baseline frequency of EMI lens.


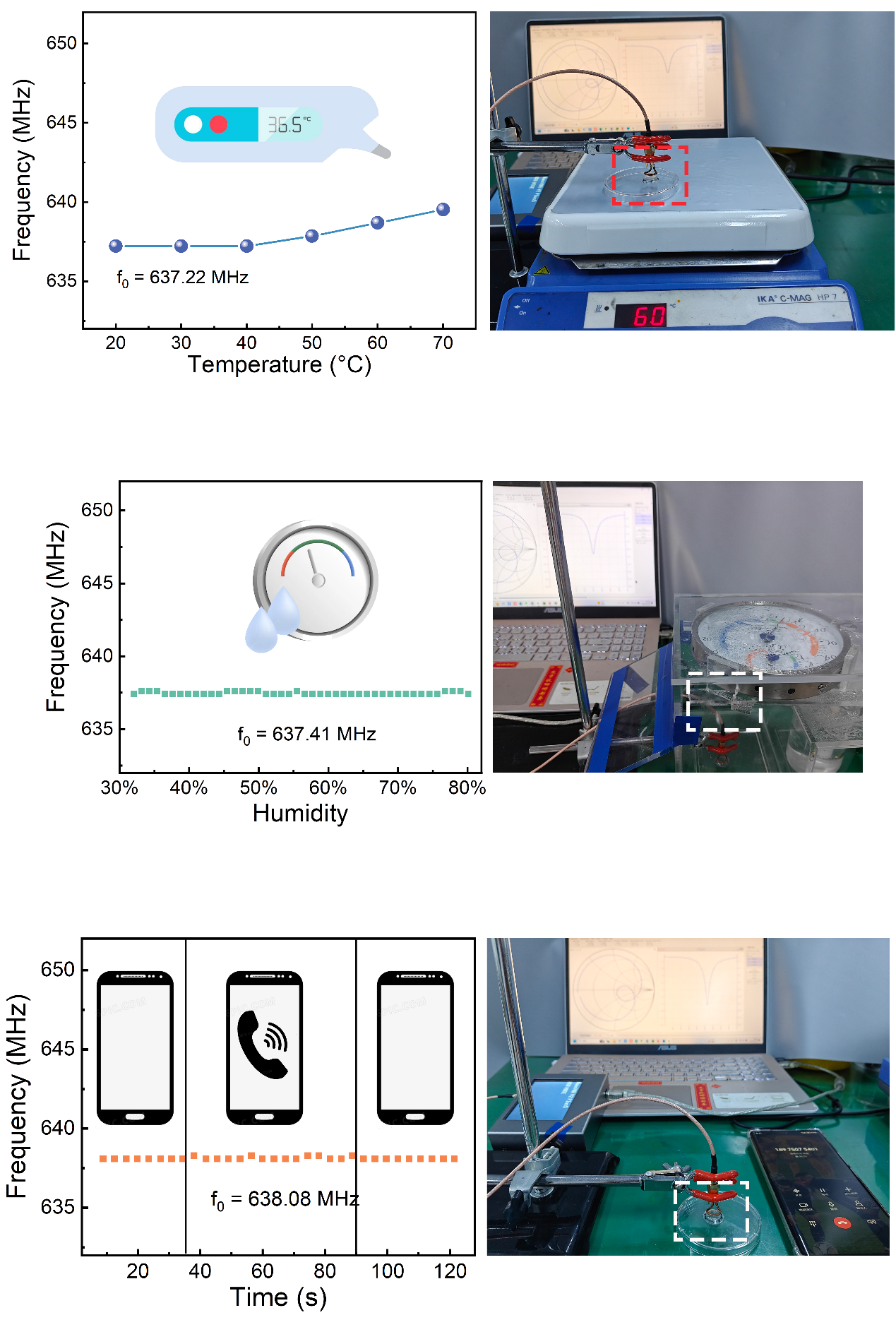


**Figure S22.** Effect of humidity on the baseline frequency of EMI lens.


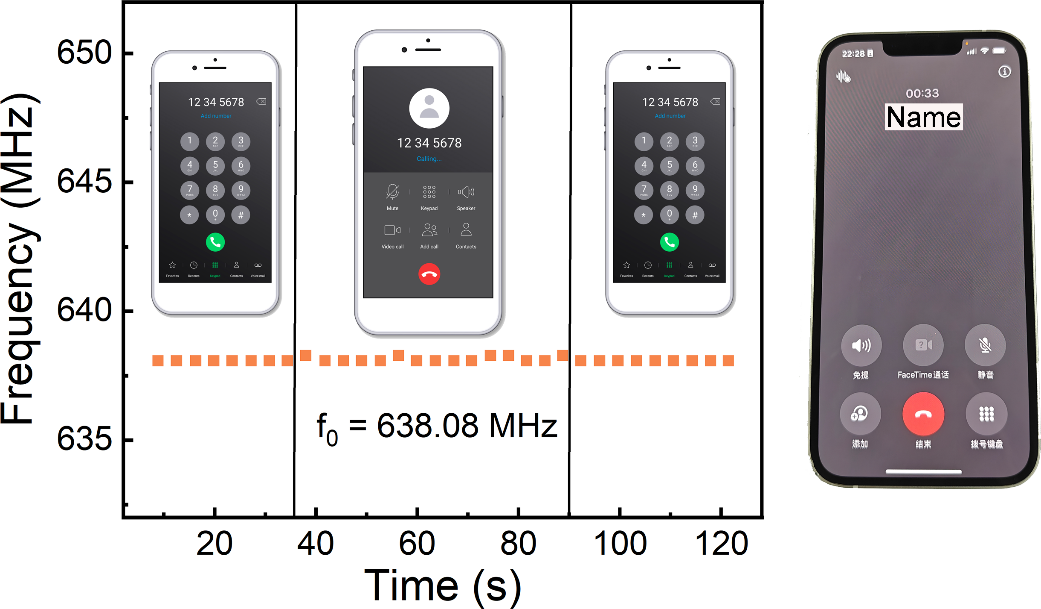


**Figure S23.** Effect of mobile phone radiation on the baseline frequency of EMI lens.

**
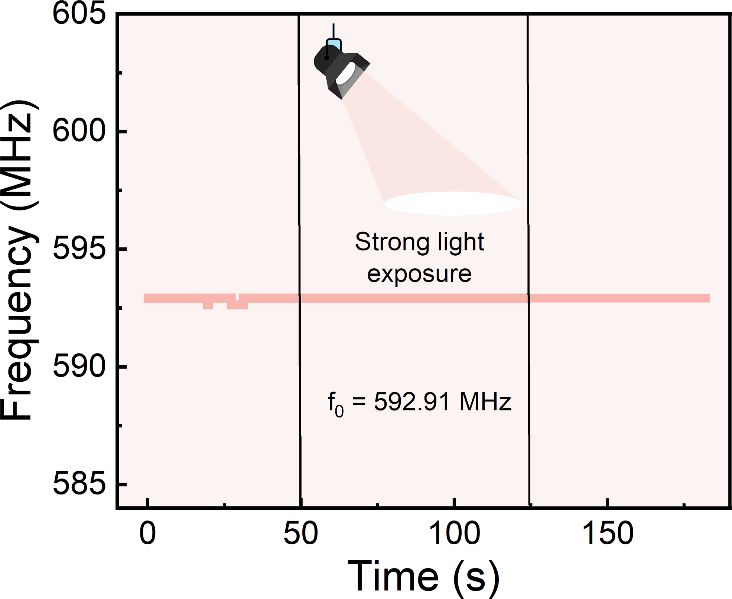
**

**Figure S24.** Effect of light conditions on the baseline frequency of EMI lens.

**
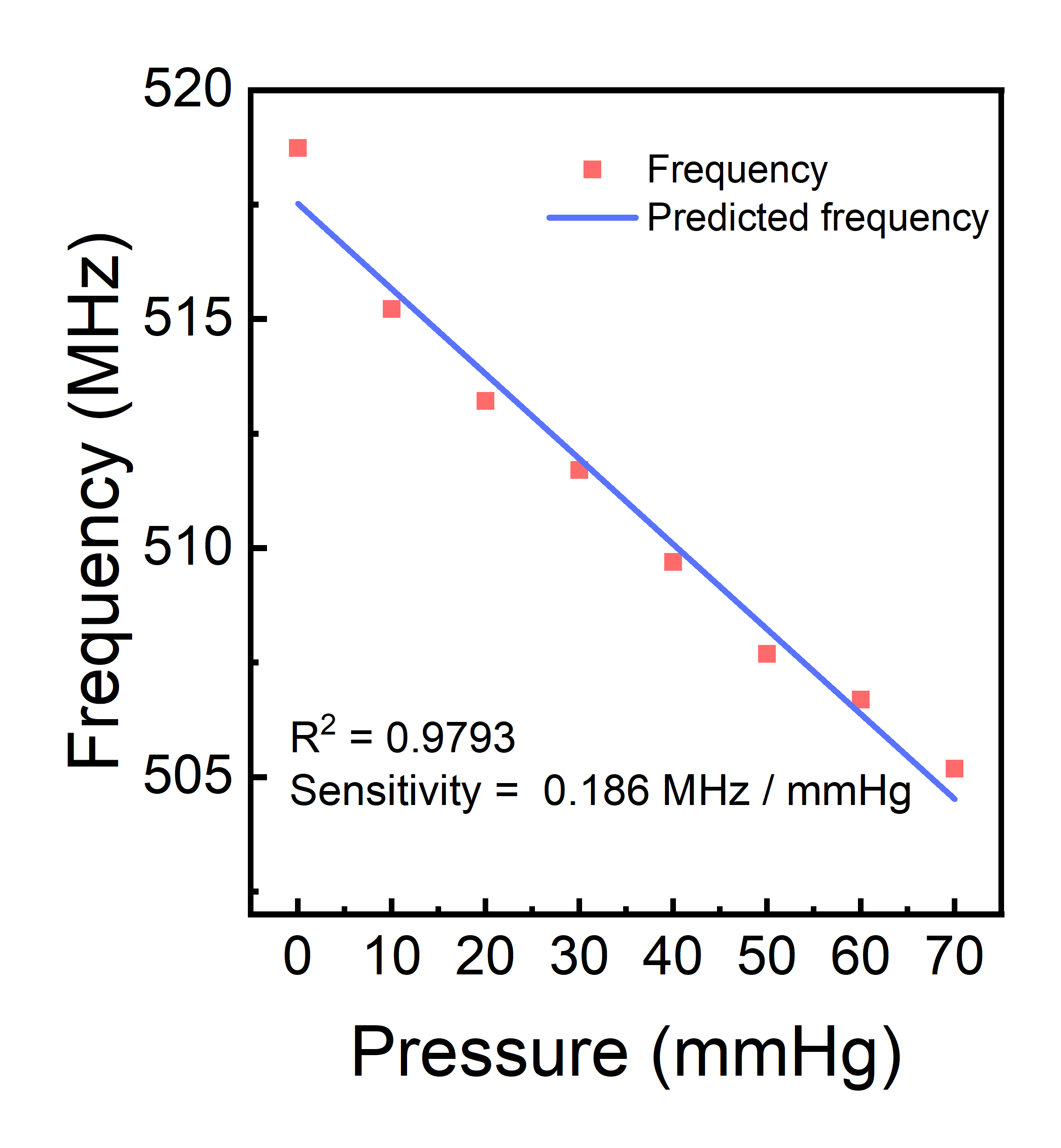
**

**Figure S25.** Linear fit curve of the frequency response of the EMI lens over a pressure range of 0-70 mmHg.


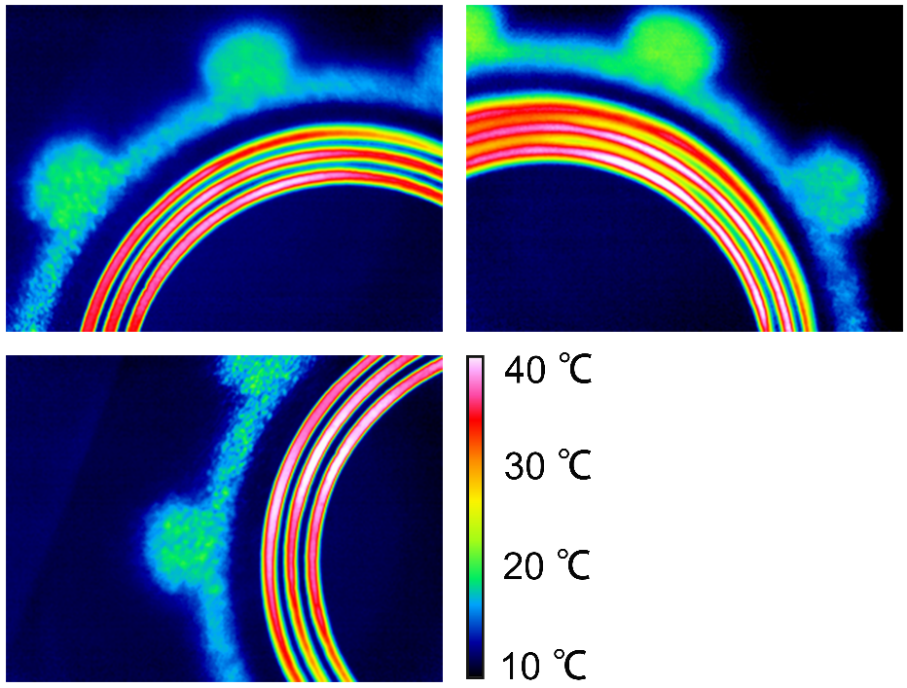


**Figure S26.** Infrared images of EMI lens in operation.


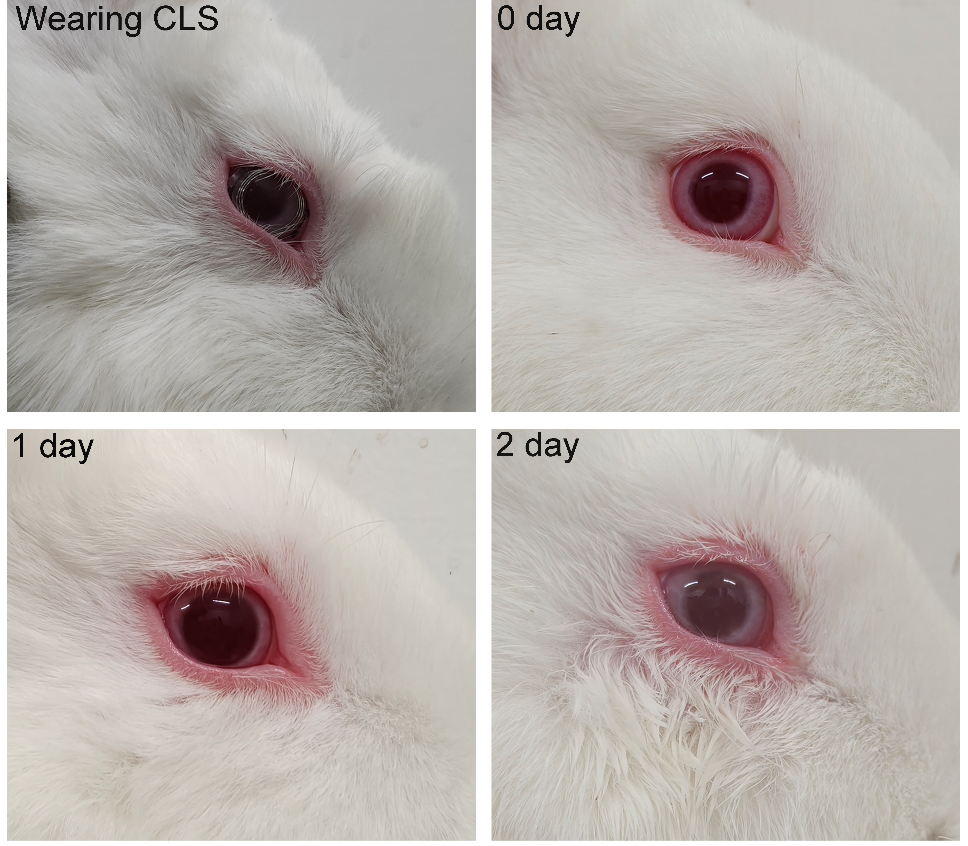


**Figure S27.** White light photographs of rabbits wearing EMI lens and eyes after 0, 1 and 2 days.


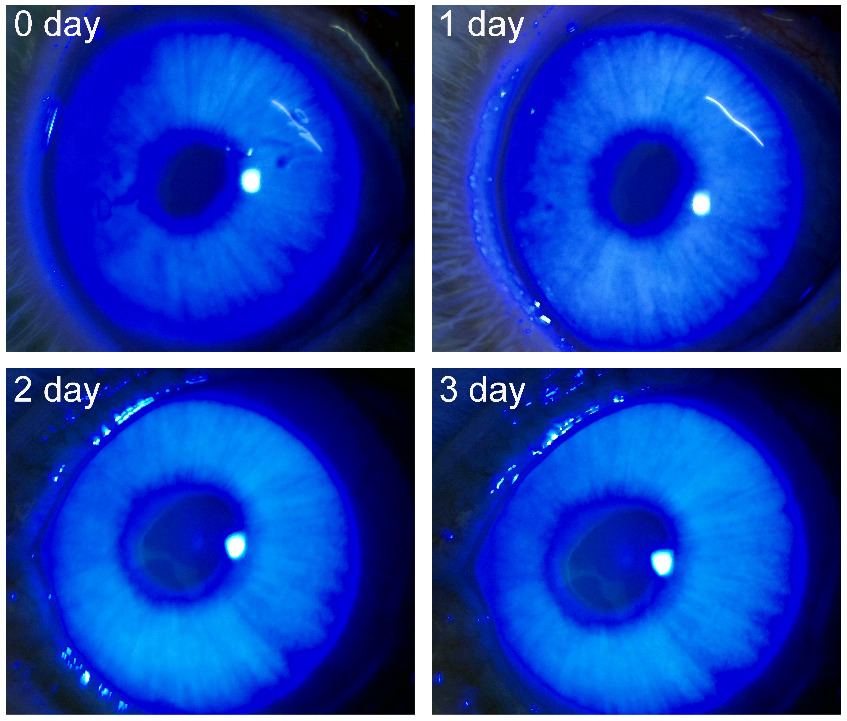


**Figure S28.** Slit-lamp photographs of the eyes of rabbits after wearing EMI lens for different days


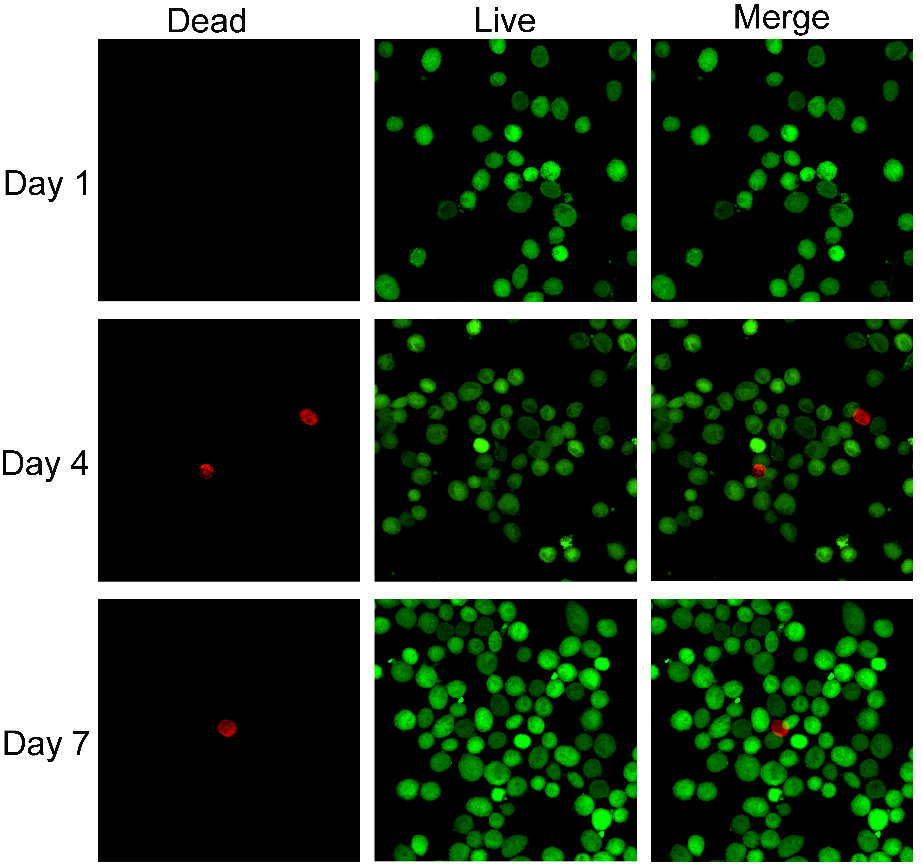


**Figure S29.** Fluorescence images of cells at different times in cytotoxicity tests. Green and red fluorescence indicate live and dead cells.


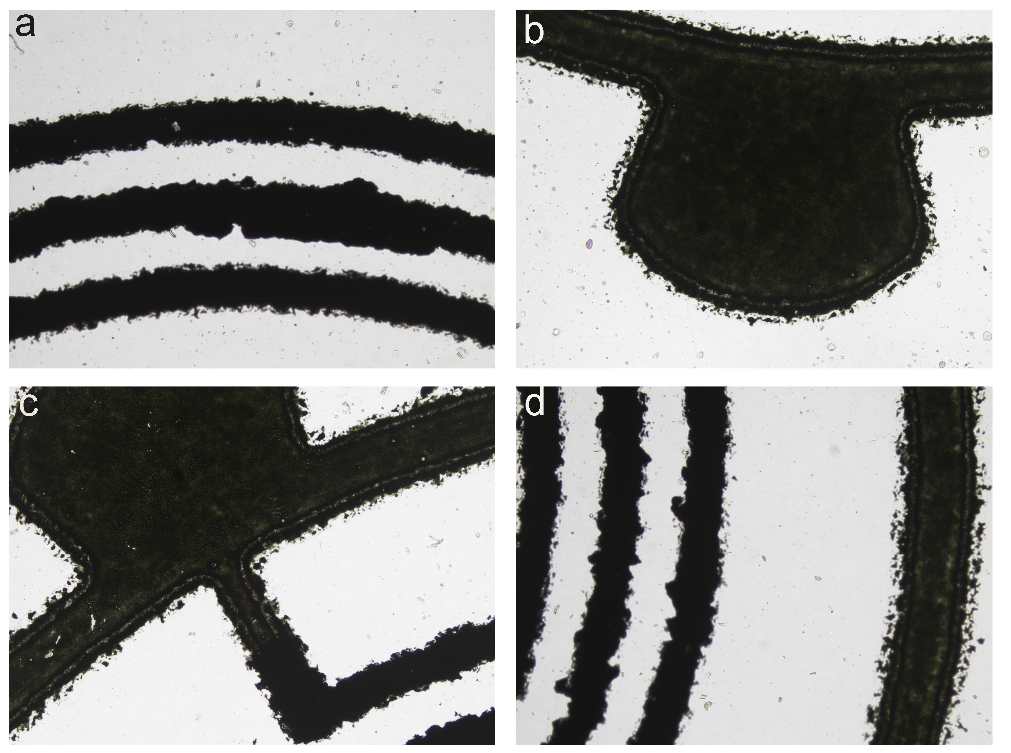


**Figure S30.** Optical images of cells and devices in cytotoxicity tests.


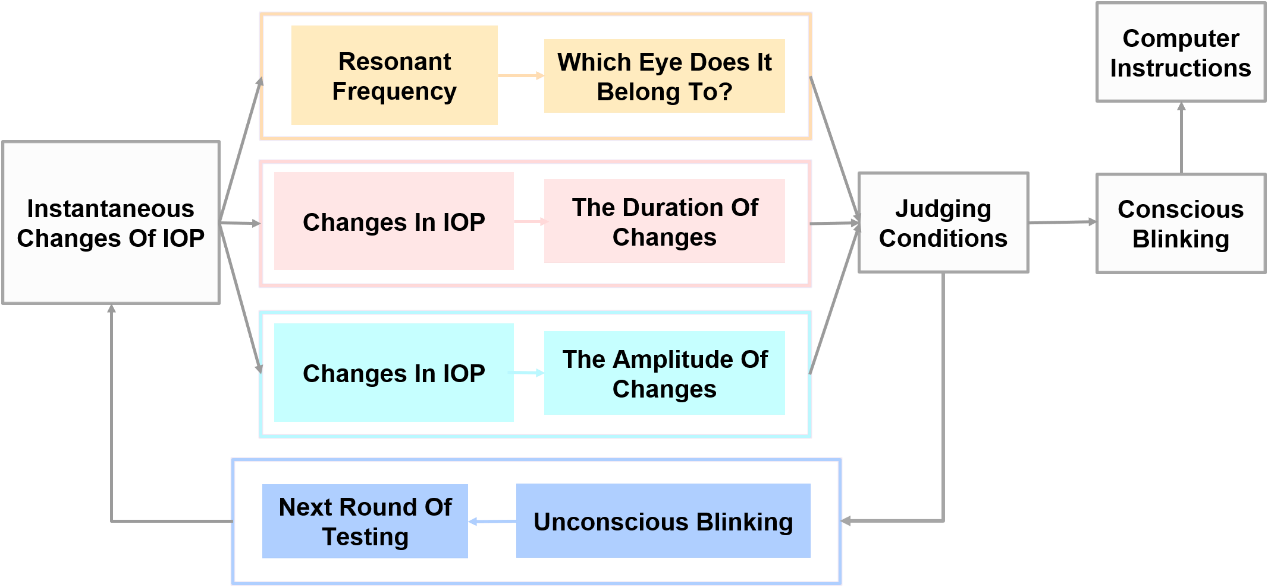


**Figure S31.** Recognition of conscious blinking and generation of computer instructions.


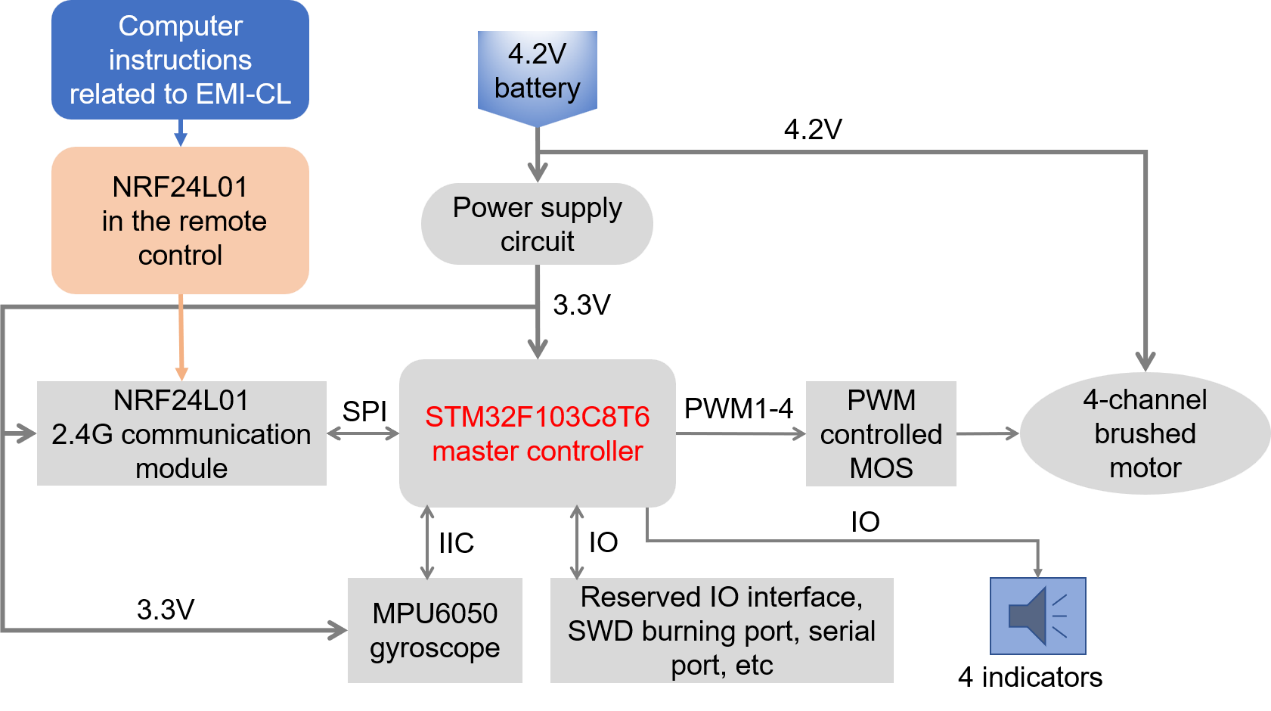


**Figure S32.** Instructions delivery and drone control flowchart.


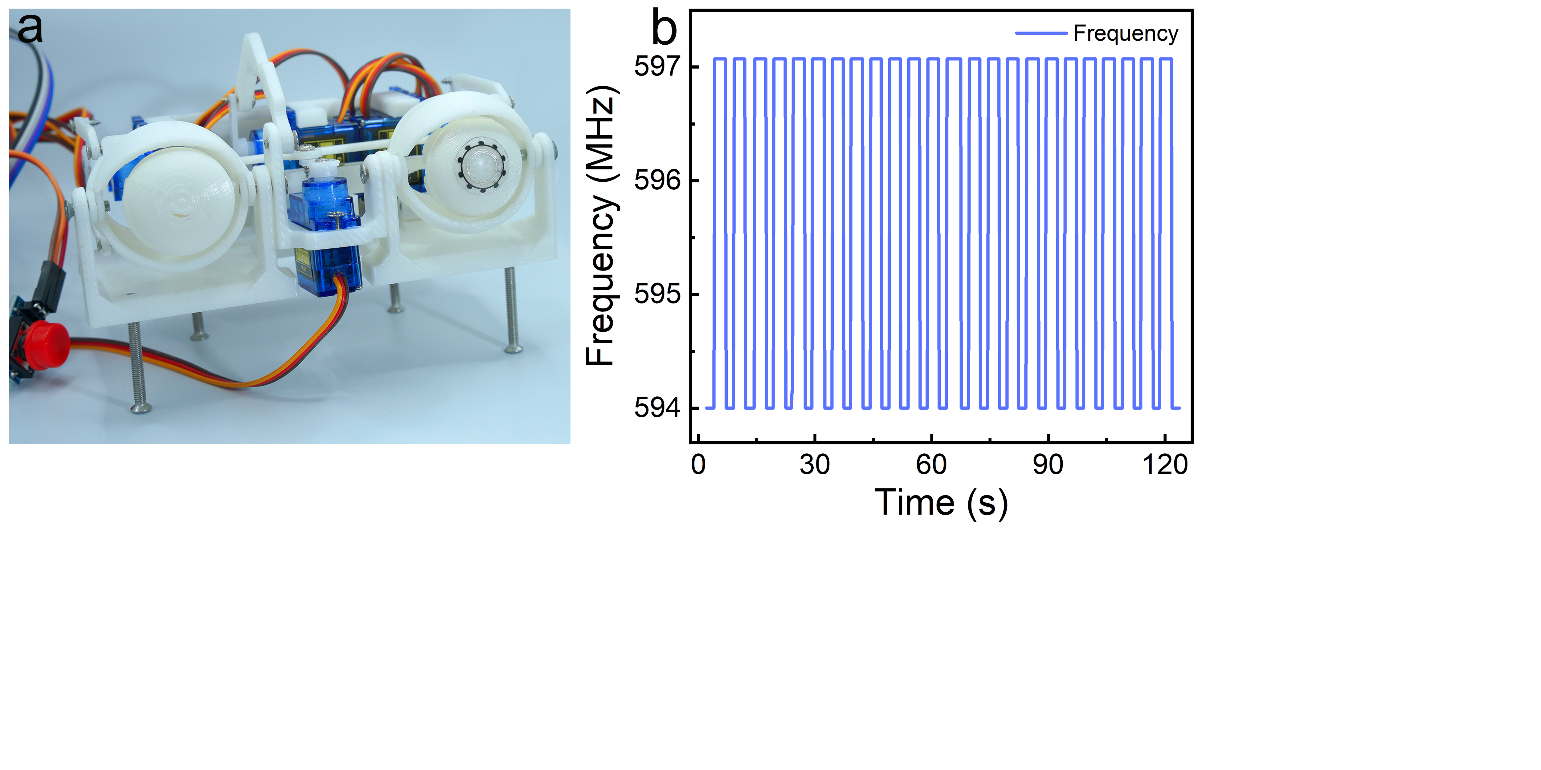


**Figure S33.** EMI lens on the eye model (a) and resonant frequency response (b) during model blinking.

**
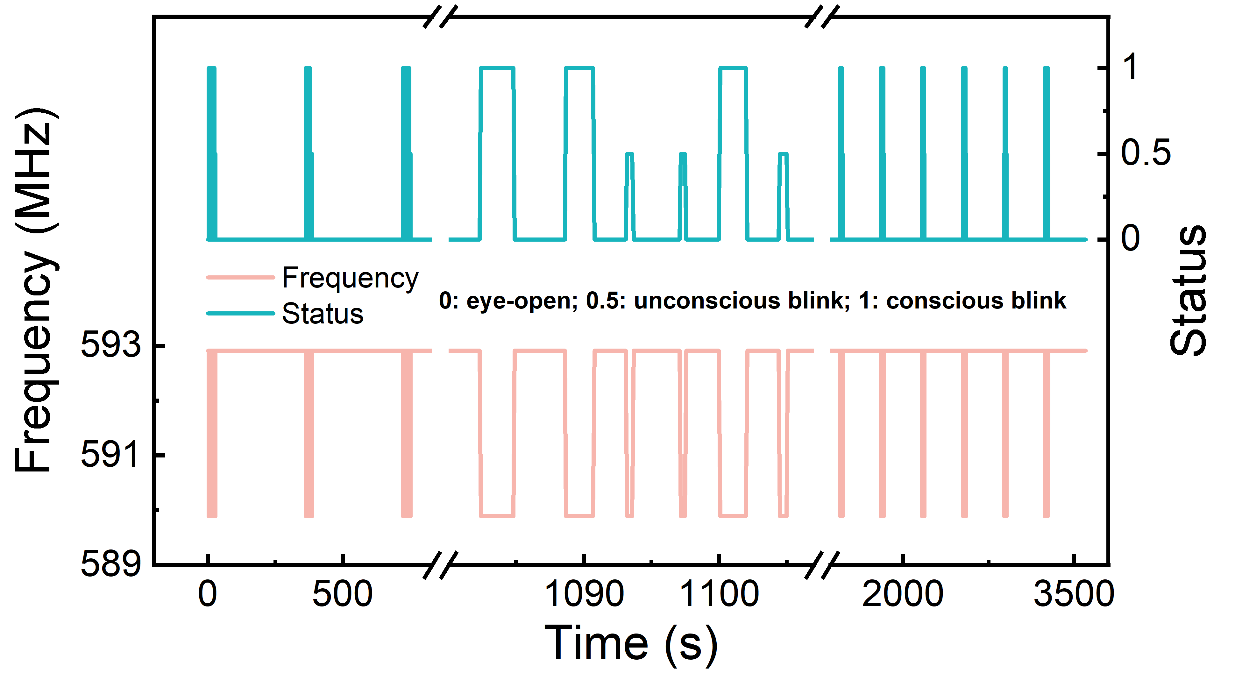
**

**Figure S34.** A one-hour experimental study on the detection of conscious and unconscious blinking.

**
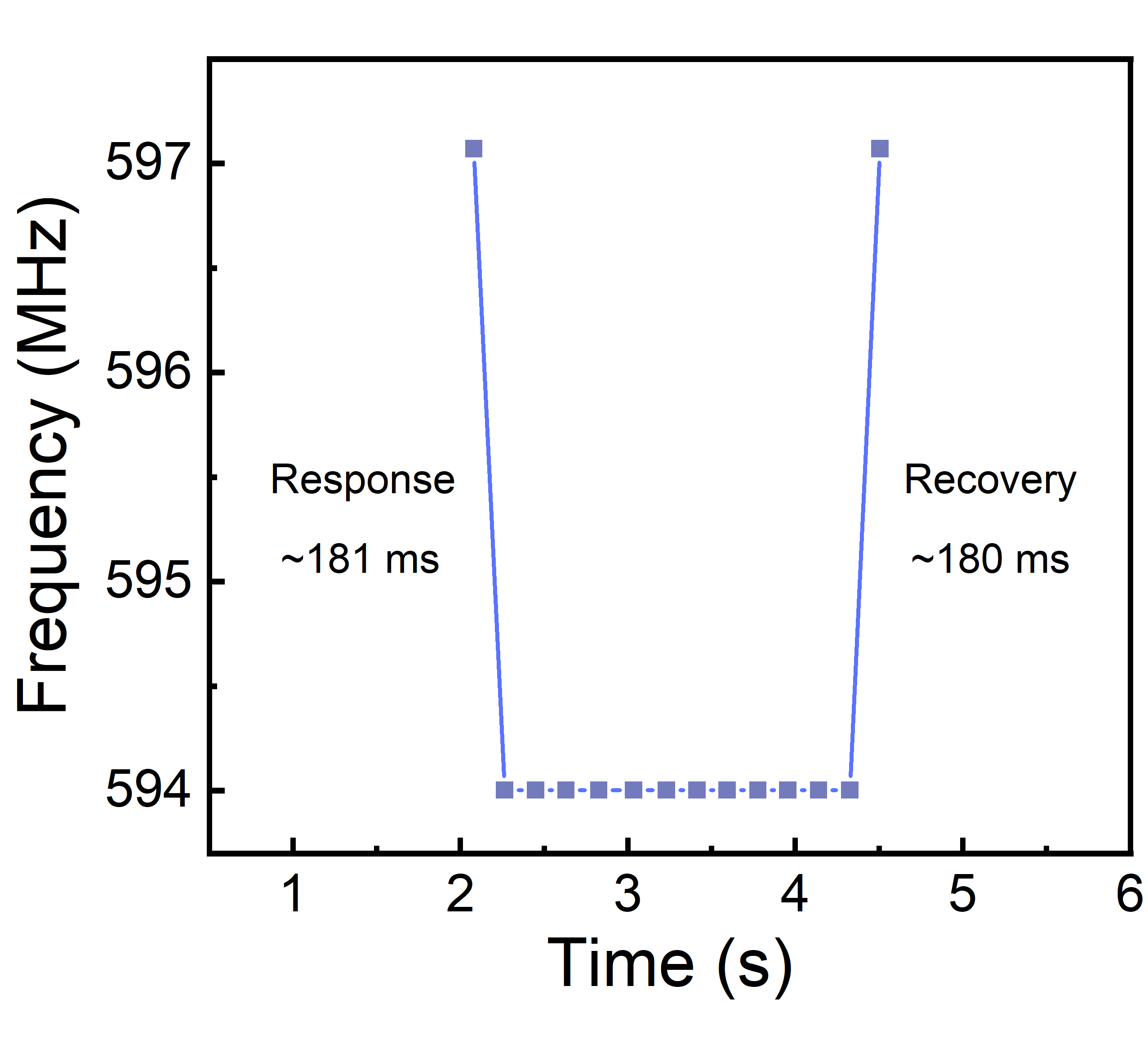
**

**Figure S35.** Frequency response and recovery time of EMI lens.


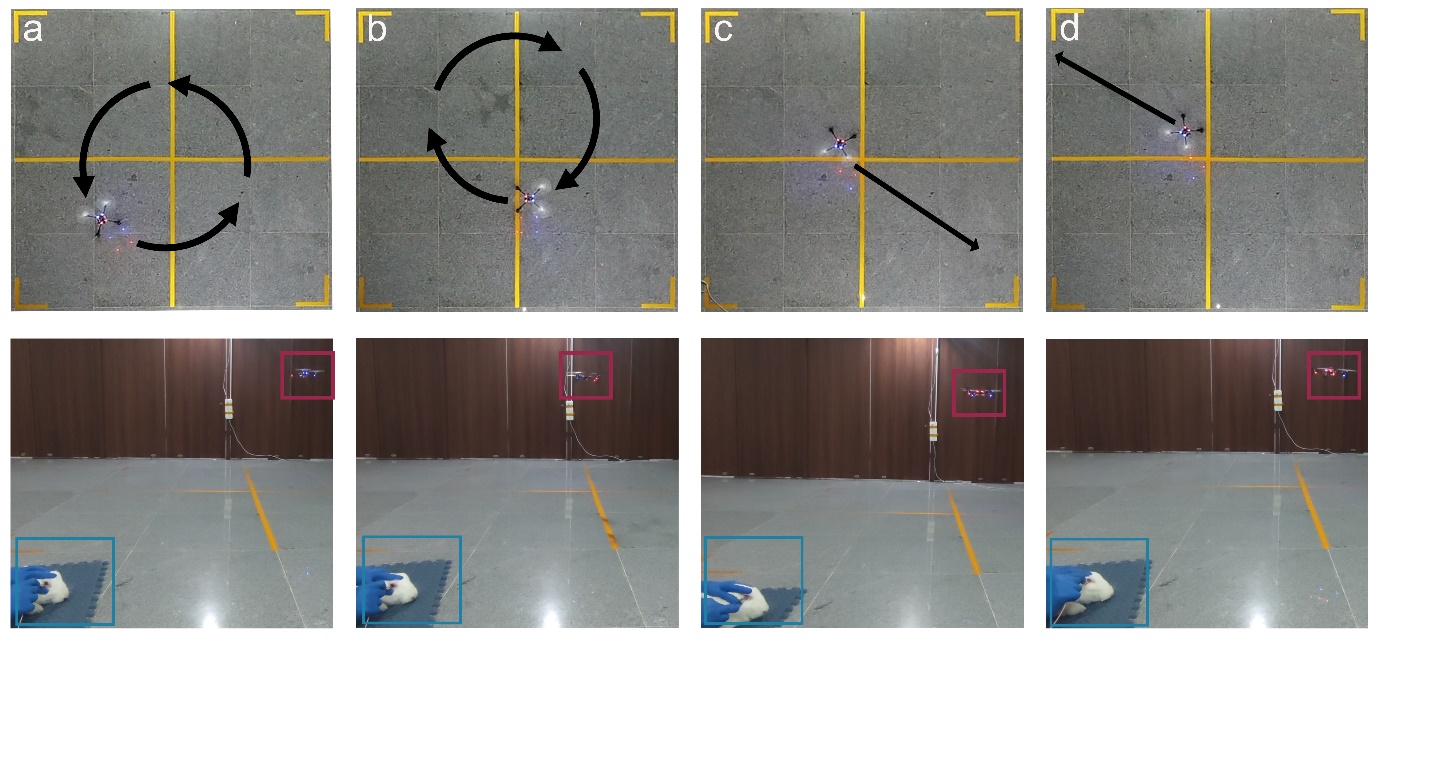


**Figure S36.** Rabbit wears EMI lens to control drone in clockwise rotation (a), counterclockwise rotation (b), forward (c) and backward (d) movement.

**Table S1.** Sensitivity comparison with reported articles.

| **Electrode material** | **Sensitivity （MHz/mmHg）** | **Reference** |
| --- | --- | --- |
| Cu | 0.112 | [11] |
| Cu | 0.057 | [12] |
| Cu | 0.068 | [13] |
| AgSEBS | 0.031 | [15] |
| liquid-metal | 0.1217 | [16] |
| Cu | 0.0045 | [17] |
| Au-Ti-Cu | 0.0468 | [18] |
| Cu-Ti-Au | 0.008 | [20] |
| Cu | 0.023 | [21] |
| **MXene** | **0.153** | **This work** |

**Table S2.** Test Range Comparison with reported articles.

| **Electrode material** | **Measuring rang (mmHg)** | **Reference** |
| --- | --- | --- |
| liquid metal | 0-50 | [10] |
| Cu | 10-50 | [11] |
| Cu | 0-55 | [12] |
| Cu | 0-30 | [13] |
| Cu | 0-50 | [14] |
| AgSEBS | 6-38 | [15] |
| liquid metal | 6-36 | [16] |
| Cu | 5-45 | [17] |
| Au-Ti-Cu | 6-61.5 | [18] |
| Graphene/AgNW | 0-50 | [19] |
| Cu, Ti-Au | 0-40 | [20] |
| Cu | 5-45 | [21] |
| **MXene** | **0-70** | **This work** |

**Table S3.** Survival index statistical table of cell in EMI lens-containing medium and control medium within 7 days.

| **Group** | **Day** | **Picture** | **Dead cells** | **Living**  **cells** | **Total cells** | **Apoptotic index** | **Survival index** | **Survival index** |
| --- | --- | --- | --- | --- | --- | --- | --- | --- |
| **1）EMI lens group** | 1D | 600-1 | 0 | 30 | 30 | 0.00% | 100.00% | **100.00%** |
|  |  | 600-2 | 0 | 29 | 29 | 0.00% | 100.00% |  |
|  |  | 600-3 | 0 | 39 | 39 | 0.00% | 100.00% |  |
|  | 4D | 600-1 | 1 | 88 | 89 | 1.12% | 98.88% | **98.28%** |
|  |  | 600-2 | 1 | 81 | 82 | 1.22% | 98.78% |  |
|  |  | 600-3 | 2 | 69 | 71 | 2.82% | 97.18% |  |
|  | 7D | 600-1 | 1 | 118 | 119 | 0.84% | 99.16% | **99.05%** |
|  |  | 600-2 | 1 | 99 | 100 | 1.00% | 99.00% |  |
|  |  | 600-3 | 1 | 97 | 98 | 1.02% | 98.98% |  |
| **2）Control group** | 1D | 600-1 | 0 | 57 | 57 | 0.00% | 100.00% | **100.00%** |
|  |  | 600-2 | 0 | 46 | 46 | 0.00% | 100.00% |  |
|  |  | 600-3 | 0 | 41 | 41 | 0.00% | 100.00% |  |

**Table S4.** Propagation rate statistical table.

|  | **OD 1** | **OD 2** | **OD 3** | **Average OD value** | **Subtract blank value** | **Propagation rate** |
| --- | --- | --- | --- | --- | --- | --- |
| Blank hole | 0.174 | 0.177 | 0.187 | 0.179 | -- | -- |
| normal group | 0.577 | 0.589 | 0.663 | 0.609 | 0.430 | **100.00%** |
| Device group-1d | 0.614 | 0.611 | 0.648 | 0.624 | 0.445 | **103.45%** |
| Device group-4d | 0.958 | 1.015 | 1.030 | 1.001 | 0.821 | **191.04%** |
| Device group-7d | 1.487 | 1.541 | 1.543 | 1.523 | 1.344 | **312.57%** |

**Movie S1. Eyeball model controlling drone movement with EMI lens.**

**Movie S2. Rabbit controlling drone movement with EMI lens.**

**Supplementary References:**

l. Li L, Liu W, Jiang K *et al.* In-Situ annealed Ti_3_C*_2_*T*_x_* MXene based all-solid-state flexible Zn-ion hybrid micro supercapacitor array with enhanced stability. *Nano-Micro Lett* 2021; **13**: 100.

2. Tian M, Lyu J, Su R *et al.* Harnessing the power of nano‐ferroelectrics: BaTiO_3_ /MXene (Ti_3_C*_2_*T*_x_*) composites for enhanced lithium storage. *Adv Energy Mater* 2024; **14**: 2401988.

3. Rong C, Su T, Li Z *et al.* Elastic properties and tensile strength of 2D Ti_3_C*_2_*T*_x_* MXene monolayers. *Nat Commun* 2024; **15**: 1566.

4. Wang F, Zhang Y, Peng Y *et al.* Continuous peroxymonosufate activation for antibiotics degradation via fluorine-free- Ti_3_C*_2_*T*_x_* -CoFe_2_O_4_ hydrogel beads: Performance, mechanism and application. *Appl Catal B Environ* 2024; **358**: 124441.

5. Wu PF, Yang YQ, Xi HY *et al.* Operando spectroscopy observation of Mo clusters‐Ti_3_C*_2_*T*_x_* catalyst/support interface's dynamic evolution in hydrogen evolution reaction. *Small* 2024; **20**: 2306716.

6. Jiang D, Cao X, Shi Y *et al.* Flexible Ti_3_C*_2_*T*_x_* MXene regulated photoelectrochemical sensing platform for sensitive monitoring of dopamine. *Adv Funct Mater* 2024; **34**: 2410546.

7. Sarycheva A, Gogotsi Y. Raman spectroscopy analysis of the structure and surface chemistry of Ti_3_C*_2_*T*_x_* MXene. *Chem Mater* 2020; **32**: 3480-3488.

8. Duan Z, Yuan M, Liu Z *et al.* An ultrasensitive Ti_3_C*_2_*T*_x_* MXene‐based soft contact lens for continuous and nondestructive intraocular pressure monitoring. *Small* 2024; **20**: 2309785.

9. Sun S, Wang J, Zhang M *et al.* MEMS ultrasonic transducers for safe, low-power and portable eye-blinking monitoring. *Microsyst Nanoeng* 2022; **8**: 63.

10. An H, Wang X, Liao Z *et al.* LC contact lens sensor for ultrasensitive intraocular pressure monitoring. *npj Flexible Electron* 2024; **8**: 53.

11. Li X, Chen W, Li H *et al.* Temperature self-compensating intelligent wireless measuring contact lens for quantitative intraocular pressure monitoring. *ACS Appl Mater Interfaces* 2024; **16**: 22522-22531.

12. Yang H, Zhu H, Liu H *et al.* Intraocular pressure monitoring smart contact lens with high environmental stability. *Adv Funct Mater* 2024; **34**: 2400722.

13. Zhu H, Yang H, Zhan L *et al.* Hydrogel-based smart contact lens for highly sensitive wireless intraocular pressure monitoring. *ACS Sens* 2022; **7**: 3014-3022.

14. Yang C, Wu Q, Liu J *et al.* Intelligent wireless theranostic contact lens for electrical sensing and regulation of intraocular pressure. *Nat Commun* 2022; **13**: 2556.

15. Zhang J, Kim K, Kim HJ *et al.* Smart soft contact lenses for continuous 24-hour monitoring of intraocular pressure in glaucoma care. *Nat Commun* 2022; **13**: 5518.

16. An H, Chen L, Liu X *et al.* High-sensitivity liquid-metal-based contact lens sensor for continuous intraocular pressure monitoring. *J Micromech Microeng* 2021; **31**: 035006.

17. Karunaratne IK, Lee CHC, Or PW *et al.* Wearable dual-element intraocular pressure contact lens sensor. *Sens Actuators, A* 2021; **321**: 112580.

18. M. Kouhani MH, Wu J, Tavakoli A *et al.* Wireless, passive strain sensor in a doughnut-shaped contact lens for continuous non-invasive self-monitoring of intraocular pressure. *Lab Chip* 2020; **20**: 332-342.

19. Kim J, Kim M, Lee M-S *et al.* Wearable smart sensor systems integrated on soft contact lenses for wireless ocular diagnostics. *Nat Commun* 2017; **8**: 14997.

20. Chen G-Z, Chan I-S, Leung LKK *et al.* Soft wearable contact lens sensor for continuous intraocular pressure monitoring. *Med Eng Phys* 2014; **36**: 1134-1139.

21. Chen G-Z, Chan I-S, Lam DCC. Capacitive contact lens sensor for continuous non-invasive intraocular pressure monitoring. *Sens Actuators, A* 2013; **203**: 112-118.
